# High-resolution livestock seasonal distribution data on the Qinghai-Tibet Plateau in 2020

**DOI:** 10.1038/s41597-023-02050-0

**Published:** 2023-03-18

**Authors:** Ning Zhan, Weihang Liu, Tao Ye, Hongda Li, Shuo Chen, Heng Ma

**Affiliations:** 1 State Key Laboratory of Earth Surface Processes and Resource Ecology (ESPRE), Beijing Normal University, Beijing, 100875 China; 2 Key Laboratory of Environmental Change and Natural Disasters, Ministry of Education, Beijing Normal University, Beijing, 100875 China; 3 Academy of Disaster Reduction and Emergency Management, Ministry of Emergency Management and Ministry of Education, Beijing, 100875 China; 4 Faculty of Geographical Science, Beijing Normal University, Beijing, 100875 China; 5 Qinghai General Station of Grassland, Xining, Qinghai 810008 China; 6 Department of Agricultural and Biological Engineering, Purdue University, West Lafayette, IN 47907 USA

**Keywords:** Environmental sciences, Natural hazards, Biogeochemistry

## Abstract

Incorporating seasonality into livestock spatial distribution is of great significance for studying the complex system interaction between climate, vegetation, water, and herder activities, associated with livestock. The Qinghai-Tibet Plateau (QTP) has the world’s most elevated pastoral area and is a hot spot for global environmental change. This study provides the spatial distribution of cattle, sheep, and livestock grazing on the warm-season and cold-season pastures at a 15 arc-second spatial resolution on the QTP. Warm/cold-season pastures were delineated by identifying the key elements that affect the seasonal distribution of grazing and combining the random forest classification model, and the average area under the receiver operating characteristic curve of the model is 0.98. Spatial disaggregation weights were derived using the prediction from a random forest model that linked county-level census livestock numbers to topography, climate, vegetation, and socioeconomic predictors. The coefficients of determination of external cross-scale validations between dasymetric mapping results and township census data range from 0.52 to 0.70. The data could provide important information for further modeling of human-environment interaction under climate change for this region.

## Background & Summary

The Qinghai-Tibetan Plateau (QTP) is the most elevated pastoral area in the world^1^, and one of China’s most important pastoral areas. It has enriched grassland resources with a total area of 1.5 million km^2^, accounting for 50.43% of the total grassland area in China^2^. Livestock grazing has important socio-ecological significance for the QTP and its surrounding areas. The main types of grazing livestock on the QTP are Yak and Tibetan sheep^3^, which are the primary sources of energy, protein, and fat for local populations^4^, supporting the survival and livelihoods of approximately 2 million pastoralists and 3 million agro-pastoralists^5^ Climate change in this region is associated with a warming and wetting trend^6^. When coupled with human activity such as fencing or overgrazing^7^, grazing livestock has put substantial stress on the grassland ecosystem and even altered the phenology of the vegetation^8,9^, thereby threatening the ecosystem stability^10,11^. Vegetation change, together with the warming trend, would consequently alter the atmosphere-hydrosphere-biosphere-lithosphere interaction^12^ and severely threaten the function of the “Asia water tower”^13,14^. Therefore, livestock grazing, as the primary means of human influence on vegetation, is key to capturing the dynamics of the human-environment interaction^15–19^. A detailed distribution of livestock data would be among the most fundamental information platforms in studying the socioeconomic, resource-environmental, livestock health, and risk assessment in the QTP, and for stakeholders to manage grassland and assign pasture for herders^20^.

Presently, most regions of QTP are using a two-season transhumance stocking system^21^. Pastures have been allocated to individual households, and livestock are grazed within the contracted and fenced household pasture parcels. Herders graze their stock on mountain slopes in the warm-season and the valleys for the cold-season^21^, but the distance of seasonal livestock migration has been limited^22^, mostly within township administrative boundaries. Such a livestock system has been the result of a set of government policies since the 1980s. Since 1985, the Chinese government has gradually implemented and established the *Household Contract Responsibility System* in pastoral areas^23^, and proposed a strategy for herders to develop from nomadic to a sedentary and semi-sedentary rotation system. Each herder household was allocated a certain area of natural pastures according to household size. Since the 1990s, seasonal pasture contracting was implemented in pastoral areas to further improve the responsibility system^23^. As pasture degradation threatened the livelihoods of residents and wildlife habitats on this plateau, the government has launched a series of ecological restoration projects and economic compensatory payment policies since 2004, and many fences have been constructed on degraded pastures to prevent new degradation^24^. This has further altered the distribution of grassland used as pastures.

Early studies on the mapping of livestock distribution were conducted at small spatial scales using direct livestock detection techniques based on moderate and high-resolution satellite imagery, either automatically or semi-automatically^25,26^. The Food and Agriculture Organization of the United Nations (FAO) proposed an approach to estimate livestock numbers within large spatial extents. Initially, stratified multiple regressions were used for linking observed livestock densities to predictors to develop the gridded livestock of the world database, GLW 2007 and GLW2 (in 2014), respectively^27^. Multiple linear regressions have also been used to identify the relationship between livestock numbers and predictors in modeling the spatial distribution of European livestock with a spatial resolution of 1 km^28^. With advances in machine learning, random forest algorithms were used to map a global 10-km livestock distribution more accurately than the previous dataset generated by multivariate regression methods^29^. In spite of the progress in modeling techniques, few studies have derived seasonal livestock distribution. Seasonal movement or transhumance is typical for nomadic or semi-sedentary livestock systems in many livestock systems around the world, to fully use environmental resources according to the seasonality of climatic conditions and grassland productivity^5,30,31^. Failing to consider seasonality in livestock distribution could bring large uncertainty in livestock system centered environmental impact or feedback analysis.

Presently, a couple of livestock distribution datasets could be useful for studies over the QTP. For instance, the GLW2 and GLW3 could be used, but suffered from coarse spatial resolution and modeling accuracy due to the lack of finer-scale local data. With support of the local data, Ye *et al*. (2019) generated a 10-km gridded carrying capacity map that approximates actual livestock distribution according to the *Forage-livestock Balance Management Approach*^32^. Li *et al*. (2021) produced a gridded livestock projection for western China with a 1 km spatial resolution by using machine learning algorithm^33^. However, these datasets don’t limit potential grazing land, nor consider seasonal livestock movement, and thus cannot reflect livestock distribution on actual seasonal pastures^34^.

Therefore, this study aims to map a high spatial resolution livestock seasonal distribution by incorporating multi-source data with machine learning, and explicitly introducing seasonal dynamics into the modeling framework. We provide a division of cold-season and warm-season pastures on the QTP, and livestock, cattle, and sheep number distribution data on each of the seasonal pastures, in dasymetric representation at a spatial resolution of 500 m.

## Methods

### Framework

**Fig. 1:**
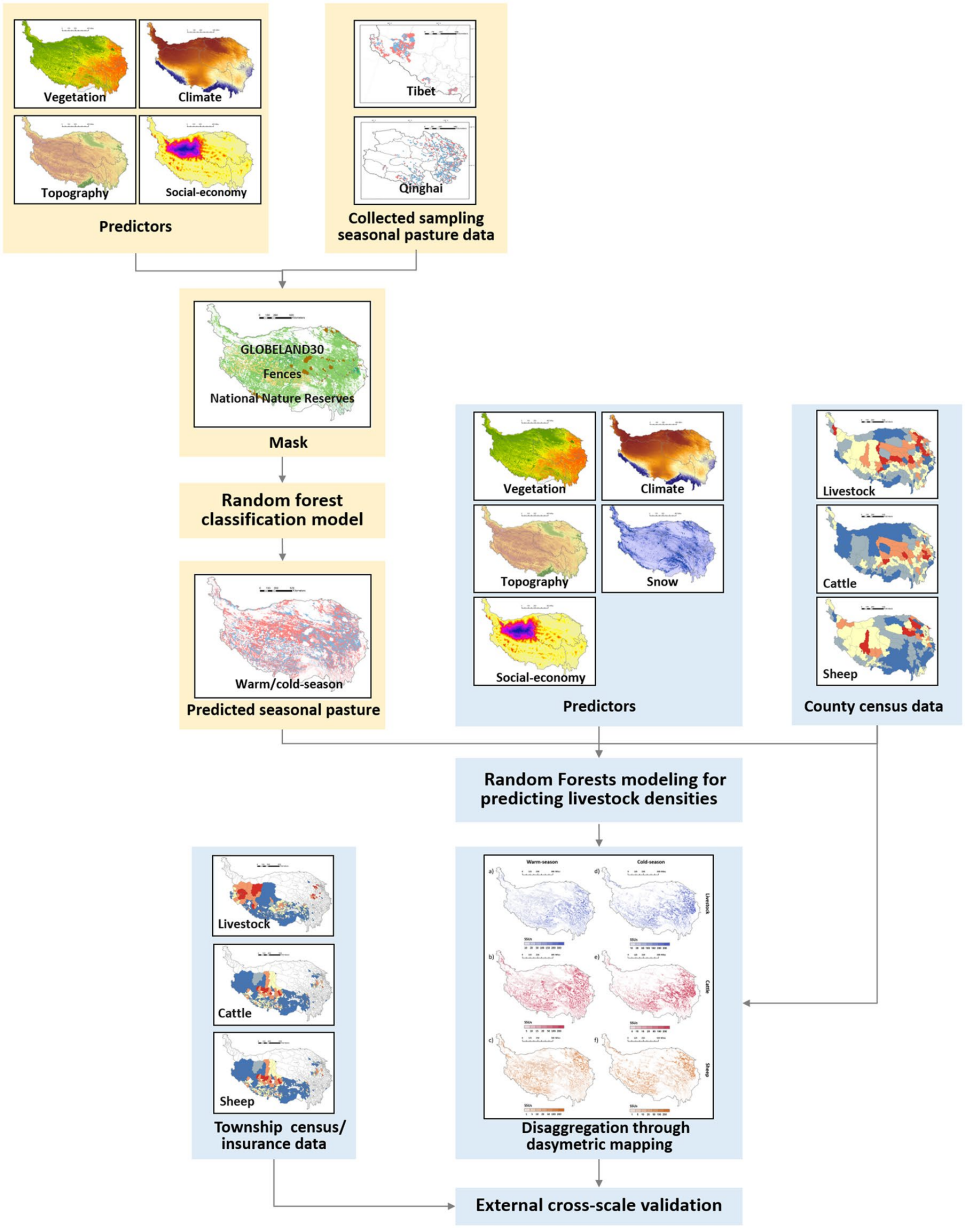
Framework of mapping livestock distributions on the Qinghai-Tibet Plateau.

In this study, a random forest classification model for predicting seasonal pastures was incorporated into the general framework of GLW3 in disaggregating livestock data, random forest modeling with the dasymetric (DA) mapping method. There are five steps to predict the seasonal distribution of livestock (Fig. 1): (1) Preparation of data and variables, (2) preparation of a pasture mask suitable for livestock grazing, (3) random forest classification modeling for predicting seasonal pastures, (4) random forest modeling for predicting livestock density distribution, and (5) dasymetric mapping for disaggregating livestock number within county boundaries.

### Preparation of data and variables

Data used in this study included data of livestock and pasture, grassland and vegetation, topography and climate, and other socioeconomic data. The list of data used in the pasture mask and the final models of the seasonal livestock distribution was shown in Table 1, and the full list of data involved in the whole modeling process was provided in Table S1.

**Table 1:** List of mask datasets and final datasets to prepare model predictors in random forest modeling.

Data type	Source Dataset	Predictor (Unit)	Description	Modeling Use	
				Seasonal pasture	Livestock number
Mask data	The 30-meter resolution global land cover data product^35^	Land cover types	Grassland/ Shrubland/ Wetland		
Topography data	The SRTM 1 Arc-Second Global DEM data^61^	DEM (m)	Digital elevation model	✓	✓
Climate data	Monthly 1-km temperature and precipitation dataset for China (2000–2017)^62^	Tmp (°C)	Annual average temperature		✓
		GStem (°C)	Average growing-season (April–Oct) temperature	✓	
		Wtem (°C)	Average snow-season (Nov–March) temperature	✓	
		GSpre (mm)	Average growing-season (April–Oct) total precipitation	✓	✓
		Wpre (mm)	Average snow-season (Nov–March) total precipitation	✓	✓
Snow data	Snow cover dataset based on multi-source remote sensing products blended on the Qinghai-Tibet Plateau (2000–2018)^63^	Snow cover days (day)	Average snow-season (Nov–March) number of snow-cover-days		✓
Vegetation data	MOD13Q1 - MODIS/Terra Vegetation Indices 16-Day L3 Global 250 m SIN Grid (2020)^64^	NDVI	Annual average maximum NDVI	✓	✓
	Vegetation map of the People’s Republic of China (1:1,000,000)^36^	Grassland type ratio (%)	The proportion of the major vegetation types		✓
Socio-economy data	1-km Global map of travel time to cities for 2015^49^	Travel time (hour)	Travel time to cities of at least 50,000 inhabitants with the shortest associated journey	✓	✓

#### Livestock and pasture data

County-level livestock census data were collected from statistical yearbooks of the study area, including six-provincial administrative regions: the Tibet Autonomous Region, Qinghai Province, Gansu Province, the Xinjiang Autonomous Region, Sichuan Province, and Yunnan Province (https://kns.cnki.net/kns8?dbcode=CYFD). These yearbooks provided the 2020 year-end number of cattle and sheep, except for Sichuan and Qinghai (year-end data of 2019). As the interannual variation of total livestock numbers was quite modest (coefficient of variation: 0.017) during 2015–2020 in the QTP, the difference was negligible for our modeling purposes. In total, livestock number data were available for 214 counties, among which data from 164 pastoral and agro-pastoral counties were used in the modeling efforts. The remaining 50 counties were identified as agricultural counties, mostly located along the eastern borders of the QTP. In these counties, livestock are mostly kept on livestock farms as opposed to open-air grazing, and their distribution could hardly be modeled with the DA approach.

Livestock data at a lower administrative level, the township level, was acquired for external model validation across different spatial scales. Census data of 36 towns in the Ngari Prefecture of the Tibet Autonomous Region was obtained from Ngari Agriculture and Animal Husbandry Bureau (https://nm.al.gov.cn/). Census data of 60 towns in Qinghai province were collected from the Agriculture and Animal Husbandry Bureau of Huangyuan County, Henan County, Maqin County, Tongde County, Zeku County, Gonghe County, Gande County, and Haiyan County (http://nynct.qinghai.gov.cn/). The insured cattle data of 654 towns and insured sheep data of 434 towns for the Tibet Autonomous Region in 2020 were obtained from the Tibet Branch of the People’s Insurance Company of China Property and Casualty (https://property.picc.com/cx_gywm/jgwd/).

**Fig. 2:**
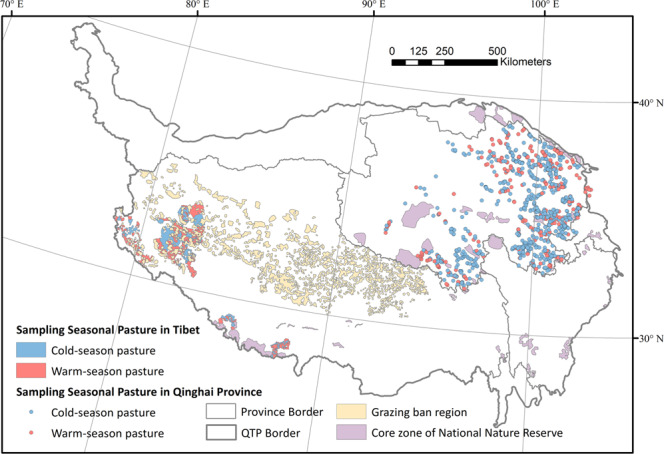
The distribution of seasonal livestock pastures sampled, the grazing ban regions, and the core zones of National Nature Reserves.

The location of seasonal pasture of Qinghai Province was obtained from the Provincial Forestry and Grassland Administration (http://lcj.qinghai.gov.cn/). The data was a part of the National Grassland Survey System operated by local survey stations affiliated to the Provincial Forestry and Grassland Administration. In total, 1365 grassland survey sample locations, with usage labels of “cold-season pasture” or “warm-season pasture” were obtained. For the Tibet Autonomous Region, the division maps of warm/cold-season pastures of 48 townships were obtained from Zhada, Geji, Jilong, and Dingjie County Forestry and Grassland Bureaus (Fig. 2). Those distribution maps were digitalized, and converted into 1 km grids. Each grid was used later as a sample point in the training of the seasonal pasture classification model.

#### Data for generating suitable pasture mask

The global land cover data offering 30 m-resolution land cover types was obtained from National Geomatics Center of China^35^. The *Vegetation Map of the People’s Republic of China*^36^, derived from a national ground-survey, gives polygon-based 11 vegetation type groupings, 55 vegetation types, and 960 vegetation groups and subgroups in China.

To generate a valid pasture boundary, we have also obtained the boundaries of National Nature Reserves from *National Nature Reserve Boundary Data* published in the Resource and Environment Science and Data Center, Chinese Academy of Science (https://www.resdc.cn/data.aspx?DATAID=272). It included 22 National Nature Reserves on the QTP. The boundary of grazing ban regions was collected from the article *Reconsidering the efficiency of grazing exclusion using fences on the Tibetan Plateau*^37^, including the area of the fence(Fig. 2). These regions are banned for livestock grazing.

#### Data for preparing random forest model predictors

The seasonal distribution of livestock is critically linked to the abundance of food sources, environmental stress, and herder activity. In light of this, topography, climate, vegetation, snow, and socioeconomic factors were all considered in preparing predictors.

Topography is the macro-controlling factor of other elements on the QTP, and digital elevation model (DEM) was used. Climate is a key factor in determining grassland types and productivity on the QTP and also includes climate harshness to grazing livestock. Monthly near-surface temperature and precipitation, and winter snow were all considered. Besides, soil moisture and evapotranspiration were also considered in training but not included in the final model. For vegetation, normalized difference vegetation index (NDVI) was used to denote vegetation productivity, and grassland type (the proportion of each major vegetation type, “Alpine Steppe”, “Alpine Meadow”, “Subalpine Shrub”, “Temperate Desert” and “Temperate Meadow”) was derived from the *Vegetation Map of the People’s Republic of China*^36^. Other information such as vegetation productivity denoted by gross primary production (GPP), net primary productivity (NPP) and vegetation coverage were considered but not included in the final model. The socioeconomic data offered population distribution, gross domestic production distribution (GDP), nighttime lights (NTL), and travel time to cities (Travel time), and only travel time was used in the final model.

### Preparation of a pasture mask suitable for livestock grazing

**Fig. 3:**
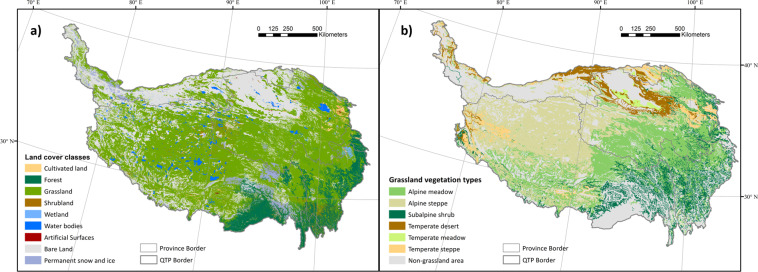
GlobeLand30 land cover classes in Qinghai-Tibet Plateau (**a**); Distribution of grassland vegetation types in Qinghai-Tibet Plateau (**b**).

**Fig. 4:**
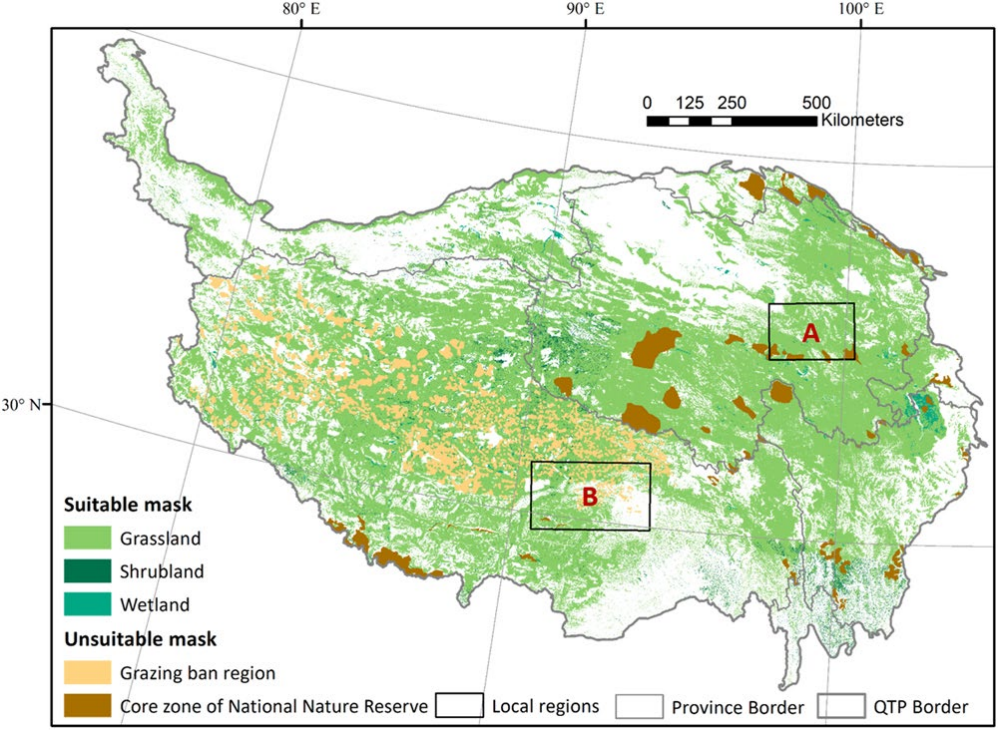
Pasture mask for livestock grazing in Qinghai-Tibet Plateau. Region A and B are two local study areas selected to display the details of livestock distribution in these two local study areas.

Land cover types suitable for grazing livestock in the QTP included grassland^38^, shrubland^39^, and wetland^40^. Correspondingly, the suitable mask was generated by fusing two datasets: the global land cover data (GlobeLand30) (Fig. 3a), and the *Vegetation Map of the People’s Republic of China*^36^ (Fig. 3b). The two datasets are in excellent agreement on the distribution of grassland, with correlation coefficients of 0.998, if summarized at the county level. In the fusion process, GlobeLand30 was used as the base mask, and the vegetation type information contained in the Vegetation Map was allocated to each 30-m pixel. Then, only the pixels with land cover types of grassland, shrubland, and wetland were kept in the mask. In addition, there are regions that livestock grazing is prohibited, according to Chinese policy, including the core zones of National Nature Reserves (https://www.resdc.cn/data.aspx?DATAID=272), and grazing ban regions^37^ (Fig. 4). These regions were excluded from the mask.

### Random forest classification modeling for predicting seasonal pastures

A random forest classification (RFC) model was used to derive the relationship between the binary response variable (warm-season pastures vs. cold-season pastures) and predictors. The model could then classify each pixel on the pasture mask into each of the warm/cold-season pastures. The binary response variable took a value of “1” if the underlying location was used as warm-season pastures, and “0” otherwise. In total, there are 69,409 pixels in Tibet Autonomous Region and 1,365 stations in Qinghai Province.

The selection of predictors has benefited from the interview of investigations at local Agriculture and Animal Husbandry Administrations, and conversations with local herder representatives during field work in 2021 and 2022. According to the interview, (1) warm-season pastures are typically situated at higher elevations, and further away from the herder’s residences, mostly on remote mountain slopes. By contrast, cold-season pastures are at relatively lower elevations, warmer in winter times, and closer to herders’ settlements, i.e., in valleys. (2) Herders generally move to warm-season pastures for grazing during the forage growing season (April–Oct) and then start to transfer to cold-season pastures for grazing around the beginning of October. (3) Due to the seasonal pasture contracting policy, each herder household has been allocated certain areas of warm-season and cold-season pastures, which might not be adjacent to each other, but must be within a township administrative boundary.

Based on the above information, predictors were prepared in following manners.

(1) As the distribution of seasonal pastures is closely related to topography, climate, forage growth, and distance to towns, our predictors included DEM, growing season (April–Oct) precipitation and temperature (GSpre, GStem), snow-season (Nov–March of the subsequent year) precipitation and temperature (Wpre, Wtem), NDVI, and the travel time to the nearest cities (Travel time) (Table 1). All predictors were resampled to 500 m.

(2) All the predictors were turned into relative values within each township boundary (1), as the allocation of seasonal pastures are totally within towns:

1 $$RV=\left({X}_{{\rm{ij}}}-\overline{{X}_{{\rm{i}}}}\right)/\overline{{X}_{{\rm{i}}}}$$

Where *RV* represents the relative value of the predictor variable, *X*_ij_ represents the raw value of the predictor variable in *j* th grid in the *i* th township, and $\overline{{X}_{i}}$ is the township average value.

The RFC model was fitted by *sklearn.Ensemble.RandomForestRegressor* toolkit^41^ in Python 3.8.8. The details of the training process were described in the codefile attached. A ten-fold internal cross-validation was applied to the raster predictors to estimate a pasture class for each pixel. The ten-fold internal cross-validation was performed by the *sklearn.model_selection.StratifiedKFold* toolkit^41^ in Python 3.8.8. The mode of the ten anticipated values for each pixel was used as the result of seasonal pasture classification. The area under the receiver operating characteristic (ROC) curve (AUC) was used to evaluate the performance of our model^42^. The AUC score ranges from 0 to 1, and accordingly prediction accuracy can be classified as excellent (0.9–1), very good (0.8–0.9), good (0.7–0.8), average (0.6–0.7), and poor (0.5–0.6).

### Random forest modeling for predicting livestock density distribution

The livestock density, equivalent to livestock number divided by the area of masked suitable lands for each county, was used as the response variable. Year-end numbers of sheep and cattle were both turned into standard sheep units (SSUs), and therefore the unit of the response variable was SSUs/km^2^. One cattle was turned into five SSUs according to the *Implementation Plan of Subsidy and Incentive Policies for Establishing Grassland Ecological Protection in Tibet Autonomous Region (2016–2020)* (http://nynct.xizang.gov.cn/).

The relationship between the natural logarithm of the response variable (livestock density) and various predictors was derived using the random forest model^43^. All predictors were resampled to 500 m. Zonal statistics, using the county polygon and the pasture mask (the warm-season pasture and the cold-season pasture together), were computed to summarize all variables to the county level for random forest modeling purposes. For each polygon, the average values of variables were taken.

The model training process tried to select a small group of predictors that enables explanatory and predicting power^44^. Before fitting the model, correlation analysis was conducted between all potential predictors (Fig. S2). Although the random forest algorithm is believed to be capable of handling multiple collinearity issues, we still cautiously tried to avoid highly correlated (|r| > 0.7^45,46^) predictors to enter the model simultaneously.

The RF model was fitted by sklearn.Ensemble.RandomForestRegressor toolkit^41^ in Python 3.8.8, and the details of the fitting could be found in the code file. A ten-fold internal cross-validation was then applied to the raster predictors to estimate a density value for each pixel. The ten-fold internal cross-validation was performed by the *sklearn.model_selection.StratifiedKFold* toolkit^41^ in Python 3.8.8. The ten predicted values were used to estimate the prediction means in each pixel. The error metrics were R^2^, mean square error (MSE), and mean absolute error (MAE). A higher R^2^, lower RMSE, and lower MAE indicate better fits between the predicted and observed values. The coefficient of variation (CV), the ratio of the standard deviation to the arithmetic mean, is employed to estimate the variability of the ten-fold internal cross-validation of each RF model^47^. A high CV value indicates a large variability of livestock density among the ten-fold internal cross-validation. On the contrary, it embodies a relatively stable livestock density.

To exclude less important predictors, we reported the cross-validation performance of models after removing each predictor with the least importance^44^, together with the partial dependence plots (PDPs)^48^ (Fig S4–S6). In the final model (Table 1), DEM reflected the topographic control effect. Annual average temperature (Tmp) and snow-season (Nov–March of the subsequent year) total precipitation (Wpre) reflects the climatological difference. The snow threat was denoted by the multi-annual average number of snow-cover-days. For the vegetation factors, NDVI was included to indicate average grassland productivity. Travel time was used to express accessibility in the RF model because it is easily interpretable and is known to be a predictive metric in research domains such as conservation, food security, trade, and population health^49^. In RF training, we also considered using the full sample and two sub-regional samples for Tibet and Qinghai to test model robustness against sample selection.

### Disaggregation through dasymetric mapping and external cross-scale validation

Dasymetric (DA) mapping is a common method for creating gridded population products, in which re-distributes census counts bounded at an administrative level onto higher-resolution spatial units^50,51^. The raster data of selected predictors were used to force the final RF model to predict the livestock/sheep/cattle distribution. Then the average density values, predicted by the final RF model, were turned into pixel-based weights in the pasture mask (the warm-season pasture and the cold-season pasture) of each county-level polygon to disaggregate county-level total numbers into pixel values. It was assumed that, livestock only distributed on cold-season pastures during the cold-season, and vice versa. For each polygon, the number of livestock per county-level polygon was multiplied by the ratio of pixel weights to the sum of pixel weights in either of the cold-season or warm-season masks. The final distribution maps of livestock numbers on the QTP were then created.

Dasymetric mapping results were aggregated by township-level polygons and compared with two sets of township-level census data for external cross-scale validation as a final measure of the data accuracy. Again, R^2^ and mean absolute error (MAE) were used as error metrics. As the DA results separated cold-season and warm-season, the external cross-scale validation process also applied to the result of both seasons.

## Data Records

Data derived with the above methods, containing the warm/cold-season spatial distribution of livestock, cattle, and sheep numbers with a spatial resolution of 15 arc-seconds (approximately 500 m), were provided in Geotiff files on the Zenodo (Link: 10.5281/zenodo.7692064)^52^. The data has a spatial extent of 73.50°E to 104.67°E and 25.99°N to 39.83°N, with 4800 rows and 2130 columns (Table 2). The coefficient of variation (CV) of livestock, cattle, and sheep density model (Table 3) and the original county statistical yearbook data in shapefiles by species are also provided (Table 4).

**Table 2:** Information of all species distribution maps provided in this study.

Filename	Description	File type	Coordinate Reference System	Spatial resolution
QTP_Da_2020_Livestock_Warm-season.tif	Livestock numbers of dasymetric product on warm-season pastures	GeoTIFF	GCS_WGS_1984	15 arc-second
QTP_Da_2020_Cattle_Warm-season.tif	Cattle numbers of dasymetric product on warm-season pastures	GeoTIFF	GCS_WGS_1984	15 arc-second
QTP_Da_2020_Sheep_Warm-season.tif	Sheep numbers of dasymetric product on warm-season pastures	GeoTIFF	GCS_WGS_1984	15 arc-second
QTP_Da_2020_Livestock_Cold-season.tif	Livestock numbers of dasymetric product on cold-season pastures	GeoTIFF	GCS_WGS_1984	15 arc-second
QTP_Da_2020_Cattle_Cold-season.tif	Cattle numbers of dasymetric product on cold-season pastures	GeoTIFF	GCS_WGS_1984	15 arc-second
QTP_Da_2020_Sheep_Cold-season.tif	Sheep numbers of dasymetric product on cold-season pastures	GeoTIFF	GCS_WGS_1984	15 arc-second

**Table 3:** Information on coefficient of variation (CV) of livestock, cattle, and sheep density model provided in this study.

Filename	Description	File type	Coordinate Reference System	Spatial resolution
QTP_CV_Livestock.tif	Coefficient of variation (CV) of livestock density model	GeoTIFF	GCS_WGS_1984	15 arc-second
QTP_CV_Cattle.tif	Coefficient of variation (CV) of cattle density model	GeoTIFF	GCS_WGS_1984	15 arc-second
QTP_CV_Sheep.tif	Coefficient of variation (CV) of sheep density model	GeoTIFF	GCS_WGS_1984	15 arc-second

**Table 4:** Information on the number of livestock at the county level in the yearbook provided in this study.

Filename	Description	Unit	File type	Coordinate Reference System	Time resolution
QTP_YB_Cattle.shp	Cattle numbers at the county-level	Ten thousand head	Shapefile	GCS_WGS_1984	2020 (2019 for Sichuan and Qinghai)
QTP_YB_Sheep.shp	Sheep numbers at the county-level	Ten thousand head	Shapefile	GCS_WGS_1984	2020 (2019 for Sichuan and Qinghai)

### Cold/warm-season pastures on the QTP

**Fig. 5:**
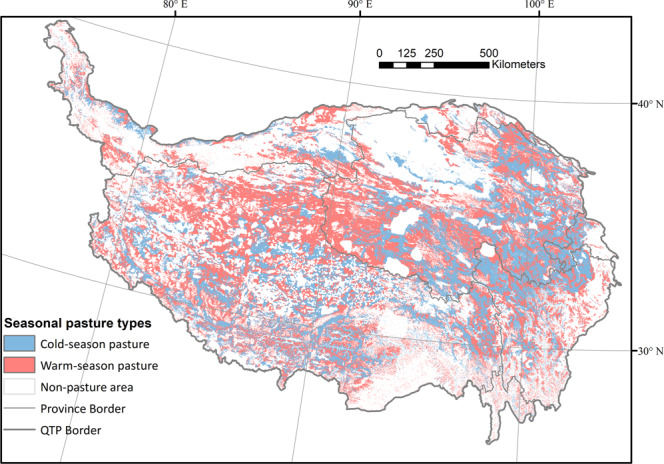
Model-derived distribution of seasonal livestock pastures on the QTP (grid size: 500 m).

The RFC model predicted the distribution of cold/warm-season pastures on the QTP (Fig. 5). The ten-fold cross-validation accuracy result of the RFC model had an average AUC of 0.98 (Fig. S1), which demonstrates that the model has an excellent ability to delineate seasonal pastures. As shown in Fig. 5, warm-season pastures are generally distributed at higher altitudes and farther away from residential settlements than cold-season pastures, which is consistent with the results of the field interview.

### Livestock distribution mapping illustration

**Fig. 6:**
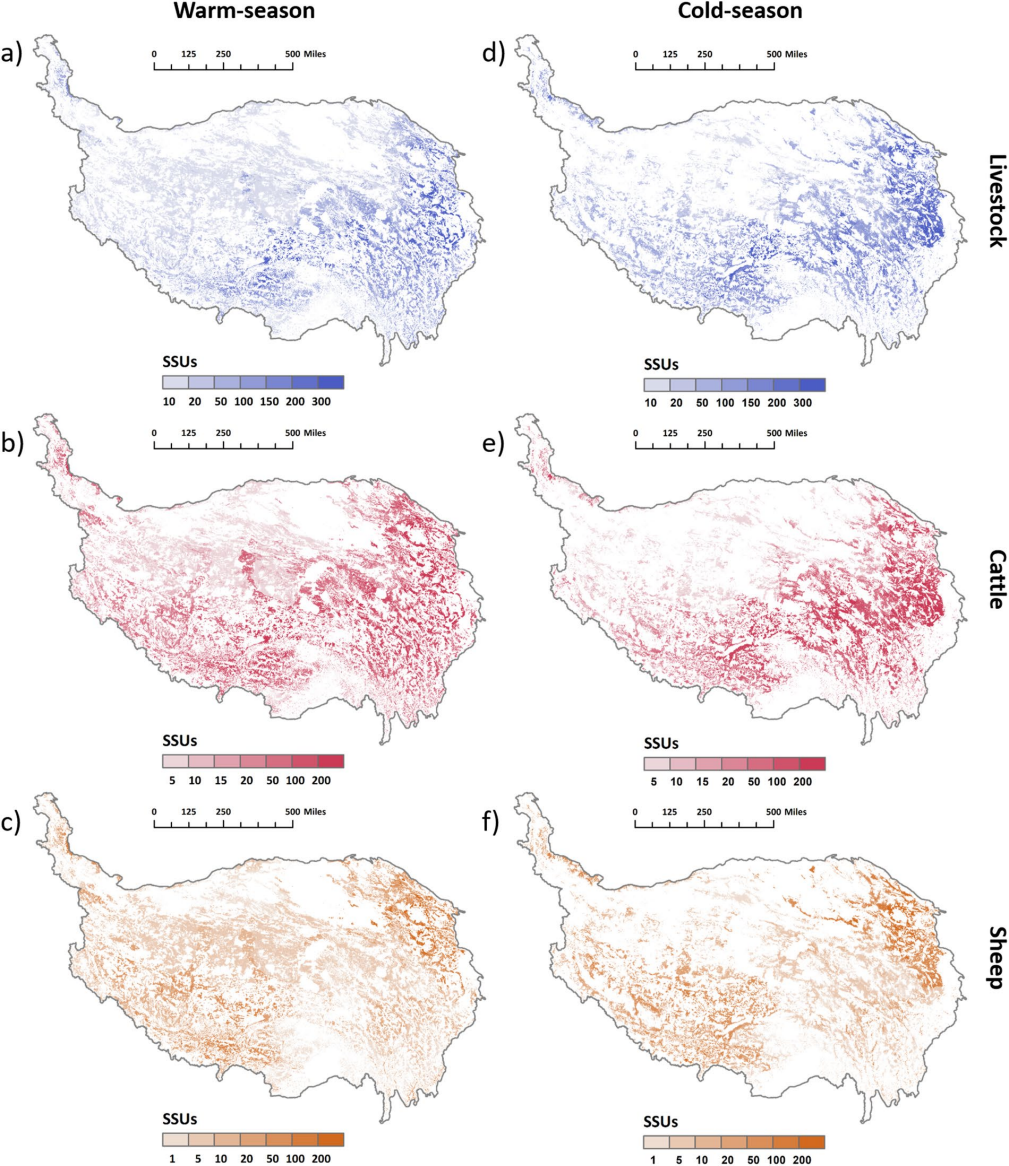
The spatial distribution of livestock numbers on the QTP. (**a**–**c**): The spatial distribution of livestock, cattle, and sheep on the warm-season pasture; (**d**–**f**): the spatial distribution of livestock, cattle, and sheep on the cold-season pasture (grid size: 500 m).

The spatial distributions of livestock numbers in cold/warm-season pastures on the QTP are illustrated in Fig. 6. The number of livestock in the seasonal pasture on the QTP decreases from southeast to northwest, with the highest number of livestock to the junction east of Qaidam Basin, north of Bayan Har Mountain, and west of Zoige Platea, where the number of livestock per grid cell (500 m) can reach more than 100 SSUs (Fig. 6). Meanwhile, livestock is densely distributed in the vast grasslands in the source regions of the Yarlung Zangbo River, Nyangqu River, and Lhasa River Region (also known as the YNL River Region^53^), the southern slope of Tanggula Mountain, with 100 to 200 SSUs in each grid. Livestock were sparsely distributed in the Qiangtang Alpine Grassland, essentially having less than 20 SSUs per grid. The number distribution of cattle in seasonal pastures showed a more obvious trend of gradually decreasing from the southeast to the northwest of the QTP. The highest cattle density in the southeast has more than 200 SSUs of cattle per grid, whereas the Qiangtang Plateau and the area around the Qaidam Basin have less than five SSUs of cattle per grid. The spatial distribution of sheep in the seasonal pasture is slightly different from the preceding two. Its distribution is primarily determined by terrain (Fig. S3). There are more sheep on each grid in the YNL River Region and the source regions of the three great rivers.

**Fig. 7:**
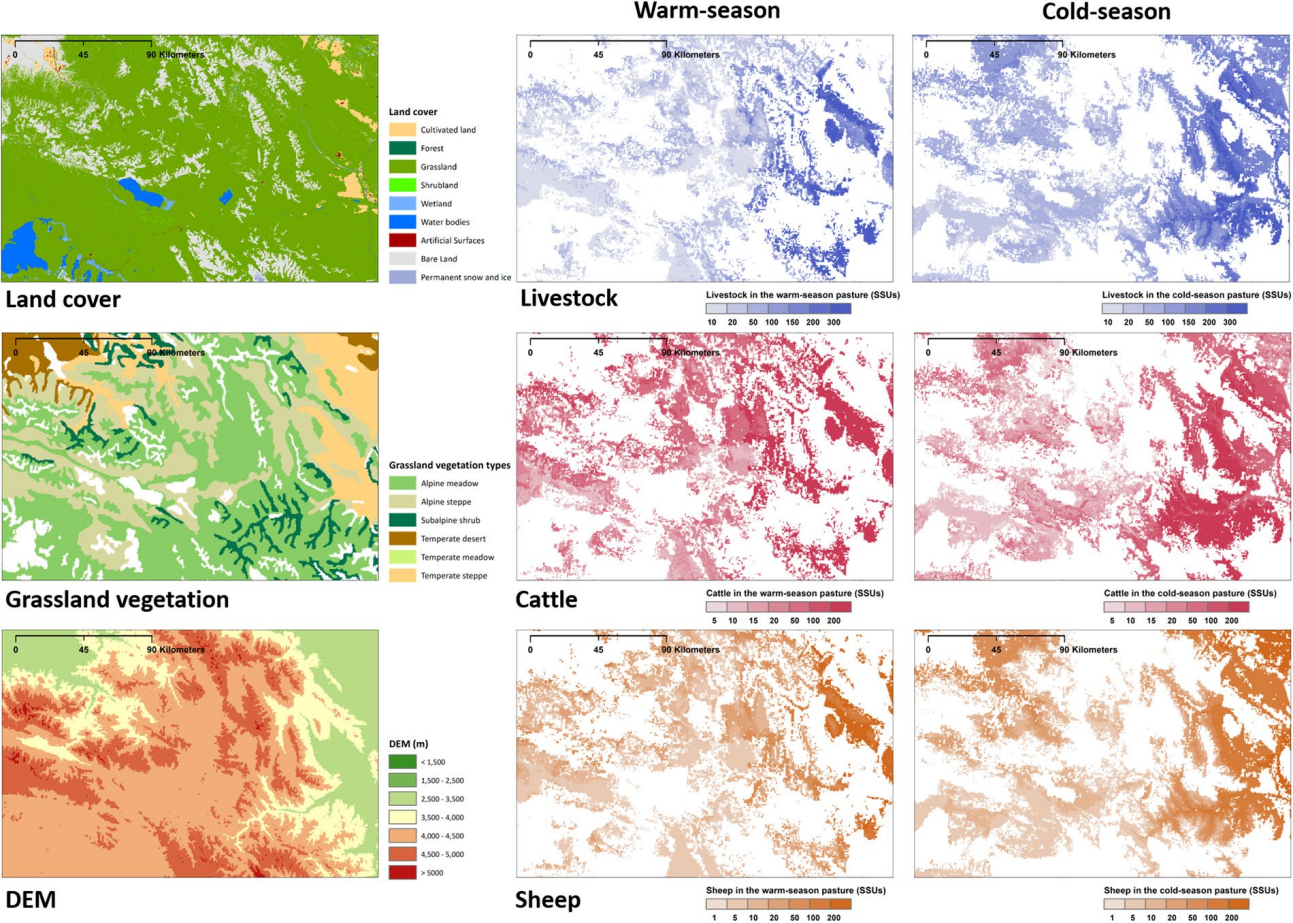
The land cover classes, grassland vegetation types, and seasonal spatial distributions of livestock in the local region A (grid size: 500 m).

**Fig. 8:**
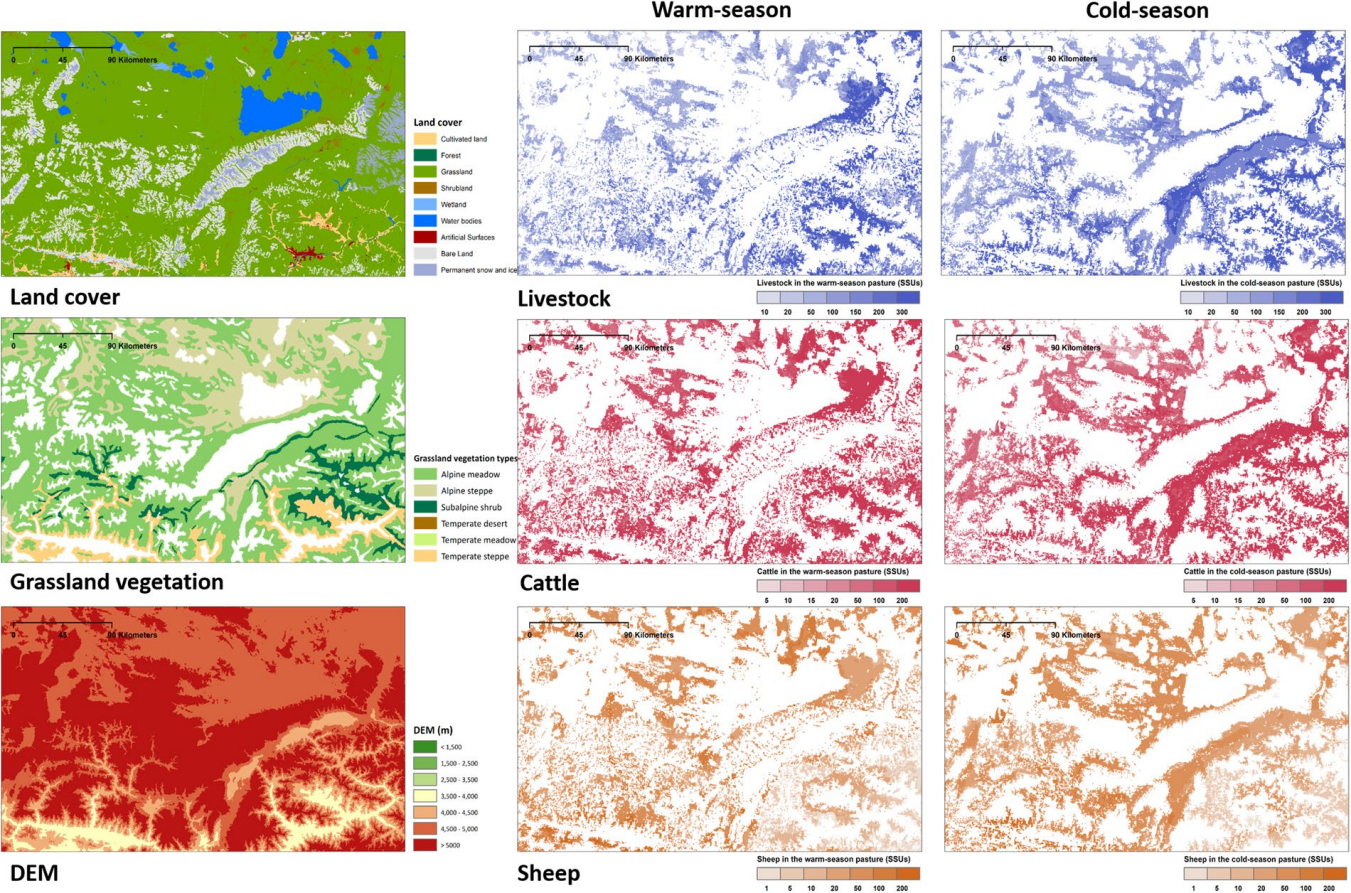
Similar to Fig. 7, but for region B.

Maps focusing on the local regions of Tibet and Qinghai Province was shown in Figs. 7 and 8, further revealing the spatial heterogeneity of livestock distribution. During the warm-season, livestock are typically found at higher elevations as opposed to lower elevations during the cold-season. Grassland vegetation plays a dominant role in the distribution of livestock and cattle, while topography is the main factor determining the distribution of sheep (Fig. S3). More livestock is distributed in places close to cultivated land and water sources, where the terrain is relatively flat and the water is relatively abundant. More livestock are distributed on the alpine meadow than on the alpine steppe when the terrain conditions are similar.

## Technical Validation

### Model internal cross-validation

The R^2^ of the internal cross-validation metric of the random forest (RF) model based on all pasture masks is between 0.68 and 0.83, indicating a reasonable goodness-of-fit (Table 5). Overall, the goodness-of-fit for sheep is better than that for cattle. The Qinghai sample results of sheep were better than the full sample (QTP), while the Tibet sample results of cattle were better than the full sample (QTP).

**Table 5:** Ten-fold cross-validation of livestock densities estimation in different regions (N represents the number of training samples (counties)).

Region	Response factor	Pasture mask	N	R^2^	MAE	MSE
QTP	Ln(Livestock density)	All pastures	164	0.700	0.510	0.250
	Ln(Cattle density)	All pastures	164	0.706	0.612	0.565
	Ln(Sheep density)	All pastures	164	0.742	0.783	0.515
Tibet	Ln(Livestock density)	All pastures	74	0.684	0.385	0.249
	Ln(Cattle density)	All pastures	74	0.809	0.401	0.274
	Ln(Sheep density)	All pastures	74	0.712	0.646	0.899
Qinghai	Ln(Livestock density)	All pastures	44	0.709	0.448	0.447
	Ln(Cattle density)	All pastures	44	0.678	0.635	0.685
	Ln(Sheep density)	All pastures	44	0.830	0.519	0.467

**Fig. 9:**
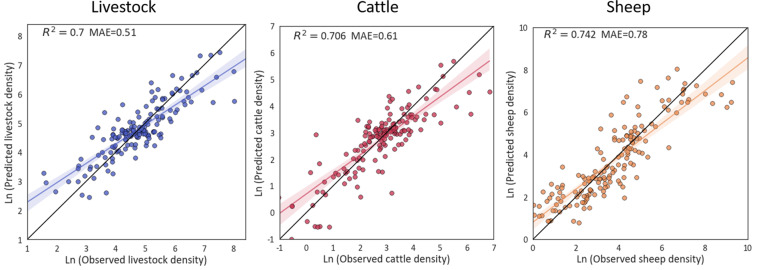
Validation of livestock densities based on ten-fold cross-validation.

As there are still 46 counties outside the Tibet Autonomous Region and Qinghai Province, results derived from the full sample of 164 counties were used for final prediction purposes. Figure 9 depicts the relationship between the predicted and observed livestock densities with respect to its natural logarithm, with each data point representing a county in the QTP.

### External cross-scale validation

**Fig. 10:**
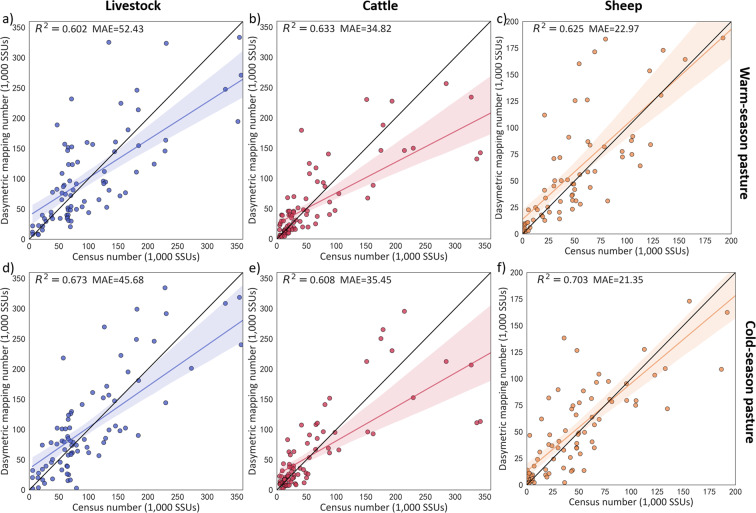
Validation results of livestock numbers between the dasymetric mapping results and township census data: (**a**–**c**): validation results of livestock, cattle, and sheep numbers on the warm-season pasture; (**b**–**f**): validation results of livestock, cattle, and sheep numbers on the cold-season pasture.

**Fig. 11:**
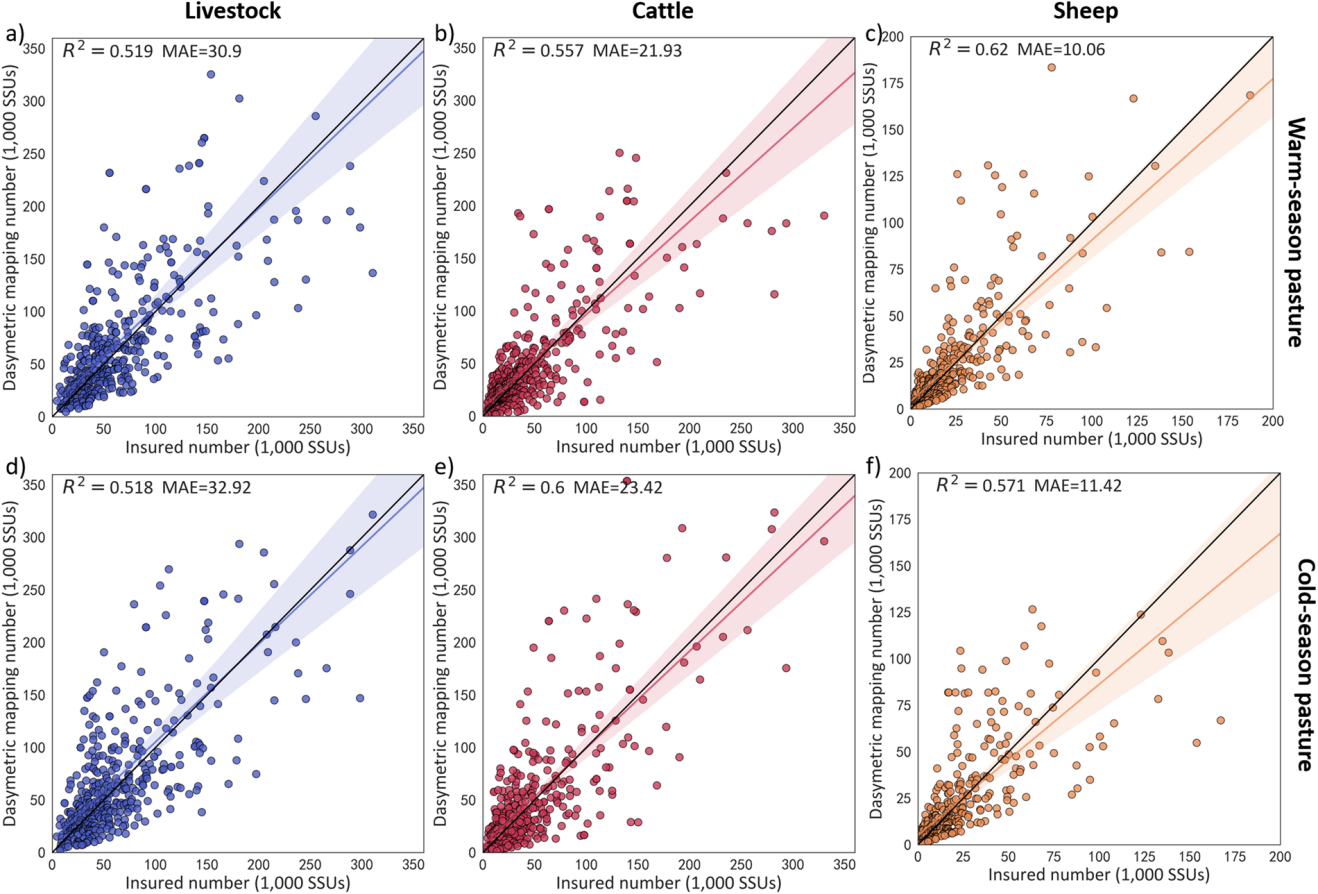
Similar to Fig. 10, but for township insured data.

External cross-scale validations were conducted between the dasymetric mapping results and township census data (Fig. 10) and township insured data (Fig. 11). All sets of validation results achieved reasonably high goodness-of-fit in terms of external cross-validation. Overall, the validation results used township-level census data are better than the Tibet insured data’s validation results. Among them, the sheep validation result using township-level census data on the cold-season pasture can reach 0.703 with a MAE of 21.35, while the validation result of livestock on the cold-season pasture using the township-level insured data can reach 0.673 with a MAE of 45.68.

## Usage Notes

The dataset generated one of the first seasonal pasture mask and provided correspondingly seasonal livestock distribution information of the QTP at the resolution of 500 m in 2020. These data sets have a wide array of potential applications in analyzing the interplay between livestock, environment, and the herder community. For instance, the dataset can be used to study the potential impact of climate change on livestock, enhancing a better understanding of sustainable livestock systems^54,55^. It could also be used for the risk assessment of natural hazards and zoonotic disease emergence^56–58^. The seasonal component is especially valuable when studying seasonal environmental stresses, i.e., snow disaster in the winter^44,59^, or heat stress during the summer^55,60^.

There are several notes for the potential users of this data. (1) We disaggregated the county-level census livestock number at the pixel level through DA mapping, and the values in each pixel reflected the livestock numbers. The users can convert to livestock density by dividing their numbers by the area of each pixel. (2) Our data only provides the spatial distribution of open-air grazing livestock. The spatial distribution for cattle and sheep in agricultural counties kept in livestock farms and fed with fodders and agricultural byproducts is much less subjected to the constraint of grassland vegetation and climate and, therefore, cannot be reasonably predicted with the DA mapping approach. (3) Users can choose the appropriate version according to their focus. For example, when studying the impact of snow hazard on livestock, the cold-season livestock distribution can be used as the base exposure. When studying the severity of heat stress on livestock in the summer, the warm-season livestock distribution could be used instead. Last but not the least, it is also worth noting that the total numbers of livestock on warm-season pastures and cold-season pastures in this study are the same at the county-level, representing livestock inventories at the county level in 2020.

Although our results have substantially improved upon the mapping of livestock on the QTP by introducing warm/cold-season difference and improving accuracy and spatial resolution, there are still uncertainties in the study results. (1) As the third pole of the world, QTP’s gridded input data, including climate, vegetation, etc. are with greater uncertainty than other regions due to the lack of ground-observation, particularly in its northwest parts. But these gridded data are the best product we could ever obtain at this stage, and have also been widely used in other studies focusing the QTP. (2) Much of our input data had a coarse resolution of 1 km, and the resampling process could have brought further uncertainty. Fortunately, the key drivers of livestock density difference were DEM and NDVI, which had spatial resolutions of 30 m and 250 m, respectively. (3) Detailed township-level census data only covered Qinghai and Tibet, but data information for other provinces, i.e., Sichuan, Gansu, and Xinjiang were absent. Fortunately, Qinghai and Tibet covered 79% grassland area of the QTP, and therefore the uncertainty of validating the model was alleviated. (4) In training the livestock distribution model, we used the county-mean of each predictor, for county-level statistics is the spatially finest official livestock number data available. Consequently, such statistical relationship derived could suffer from uncertainty should the value of predictors vary largely within counties. Our pasture mask helped reduce heterogeneity by excluding non-pasture pixels, and the within-county standard deviations of predictors were relatively small as compared to their corresponding means (Table S2). (5) Random forest model derived different runs when fed with random seeds. We used the coefficient of variation (CV) of density estimates derived from the 10-fold cross-validation process to denote the agreement/disagreement of model runs (Fig. S7). The users could judge the quality of the model results based on the CV layer.

## Supplementary information

Supplementary information

## Data Availability

The code in this study is fully operational under Python 3.8.8, and the key packages were contained in the *sklearn.Ensemble.RandomForestRegressor* and the *sklearn.model_selection.StratifiedKFold* toolkit^41^ in Python 3.8.8. The code can be found on GitHub (https://github.com/NingZhan1978/High-resolution-livestock-seasonal-distribution-data-on-the-Qinghai-Tibet-Plateau-in-2020.git).
