# Supplementary material for: High-resolution livestock seasonal distribution data on the Qinghai-Tibet Plateau in 2020
Source: Sci Data. 2023 Mar 18;10:142. doi: 10.1038/s41597-023-02050-0 (PMC10023705; doi:10.1038/s41597-023-02050-0)
Supplement: Supplementary file 1 — Supplementary information [file 41597_2023_2050_MOESM1_ESM.pdf]

Additional Formatting Information

Supplementary information

Catalogue

Supplementary Table.S1 Full list of final datasets to prepare model predictors in random forest modeling..2
Supplementary Table.S2 The mean /standard deviation of final datasets to prepare model predictors in random forest modeling within each county census unit of the QTP..3

Supplementary Fig.S1 Ten-fold cross-validation results of the random forest classification model. 7
Supplementary Fig.S2 Correlation between livestock density and covariates.................7
Supplementary Fig.S3 a) Contribution of covariates to livestock density estimation; b) Contribution of covariates to cattle density estimation; c) Contribution of covariates to sheep density estimation. 7
Supplementary Fig.S4 Response of covariates to livestock densities given by the partial dependence plot..8
Supplementary Fig.S5 Response of covariates to cattle density given by the partial dependence plot..9
Supplementary Fig.S6 Response of covariates to sheep density given by the partial dependence plot..10
Supplementary Fig.S7 Coefficient of variation (CV) of livestock, cattle, and sheep density model (The CV of the ten-fold internal cross-validation results). 10

Supplementary Table.S1 Full list of final datasets to prepare model predictors in random forest modeling

Data type	Source Dataset	Predictor (Unit)	Description	Modeling Use	
				Seasonal pasture	Livestock number
Topography data	The SRTM 1 Arc-Second Global DEM data[1]	DEM (m)	Digital elevation model	✓	✓
	Global 3 Arc-Second Terrain Slope and Aspect Data[2]	Slope (%)	Slope		✓
Climate data	Monthly 1-km temperature and precipitation dataset for China (2000 -2017)[3]	Tmax (°C)	Average annual maximum temperature		✓
		Tmin (°C)	Average annual minimum temperature		✓
		Tmp (°C)	Average annual mean temperature		✓
		GStem (°C)	Average annual grassland growing-season (April–Oct) mean temperature	✓	
		Wtem (°C)	Average annual snow-season (Nov–March) mean temperature	✓	
		GSpre (mm)	Average annual grassland growing-season (April–Oct) total precipitation	✓	✓
		Wpre (mm)	Average annual snow-season (Nov–March) total precipitation	✓	✓
		R95p (mm)	Multi-annual average total precipitation when daily precipitation amount ＞95th percentile (R95p)		✓
	Daily 25 km standardized soil moisture index (2000-2018) from CN05.1 meteorological data[4]	SSMI	Multi-annual average standardized soil moisture index in Jun/ Jul/ Aug/ Sept		✓
	MOD16A2 v006 - MODIS/Terra Net Evapotranspiration 8-Day L4 Global 500 m SIN Grid (2000–2020)[5]	ET (kg/m2/8day)	Average annual total Evapotranspiration (ET) each year		✓
Snow data	Long-term series of daily 25 km snow depth dataset in China (2000–2020)[6]	Snow depth (mm)	Multi-annual average snow-season snow depth		✓
	Snow cover dataset based on multi-source remote sensing products blended on the Qinghai-Tibet Plateau (2000–2018) [7]	Snow cover days (day)	The multi-annual average number of snow-cover-day in the snow-season (Nov–March)		✓
Vegetation data	MOD17A3H v006 - MODIS/Terra Net Primary Productivity 8-Day L4 Global 500 m SIN Grid (2020)[8]	GPP (kg C/m²)	Annual maximum gross primary production (GPP)		✓
	MOD17A3H v006 - MODIS/Terra Gross Primary Productivity 8-Day L4 Global 500 m SIN Grid (2020)[9]	NPP (kg C/m²)	Annual maximum net primary productivity (NPP)		✓

Data type	Source Dataset	Predictor （Unit）	Description	Modeling Use	
				Seasonal pasture	Livestock number
	MOD13Q1 - MODIS/Terra Vegetation Indices 16-Day L3 Global 250 m SIN Grid (2020)[10]	NDVI	Annual maximum Normalized difference vegetation index (NDVI)	✓	✓
	2013 1-km plant coverage (%) of the grasslands on the Tibetan Plateau[11]	Grassland plant coverage (%)	Plant coverage (%) of the grasslands		✓
	Vegetation map of the People's Republic of China (1:1,000,000)[12]	Grassland type coverage ratio (%)	The proportion of each major vegetation type （"Alpine Steppe", "Alpine Meadow", "Subalpine Shrub", "Temperate Desert" and "Temperate Meadow")		✓
Social-economy data	China GDP spatial distribution 1-km grid dataset[13]	GDP (10,000 RMB/km^2)	Gross domestic product (GDP)		✓
	The Defense Meteorological Satellite Program (DMSP) - Operational Linescan System (OLS) 1-km nighttime light (NTL) data[14]	NTL (nano-Wcm^{-2} sr^{-1})	Average nighttime lights (NTL) of the year 2020		✓
	2019 LandScan 1-km Global population[15]	Population	The population count for the year 2019		✓
	1-km Global map of travel time to cities for 2015[16]	Travel time (hour)	Travel time to cities of at least 50,000 inhabitants with the shortest associated journey	✓	✓

Supplementary Table.S2 The mean /standard deviation of final datasets to prepare model predictors in random forest modeling within each county census unit of the QTP.

Census Unit	DEM		TEM		GSpre		Wpre		Snow cover days		NDV		Travel time	
	Mean	STD	Mean	STD	Mean	STD	Mean	STD	Mean	STD	Mean	STD	Mean	STD
Fugong County	3216.86	754.87	10.17	4.63	782.91	201.25	192.10	37.24	11.80	8.34	0.85	0.06	1018.41	361.49
Ning Langyizu Autonomous County	3105.70	531.01	9.61	3.21	698.62	22.87	111.07	8.16	2.67	1.91	0.78	0.07	860.85	313.32
Wei Xilisuzu Autonomous County	3323.07	643.97	8.17	4.23	706.39	95.86	173.03	31.75	14.15	13.27	0.79	0.09	1036.19	370.64
Deqin County	4128.48	836.64	3.60	5.48	590.79	75.77	116.80	26.77	24.69	23.80	0.61	0.18	1212.91	515.04
Gong Shandonglongzunuzu Autonomous County	3686.72	641.80	5.10	3.89	718.98	173.05	159.54	29.45	30.54	18.06	0.68	0.14	1575.49	444.19
Yecheng County	3157.77	945.20	2.12	6.27	19.16	3.97	5.60	2.16	29.60	23.73	0.37	0.16	835.17	1465.83
Cele County	3049.30	1250.62	1.99	7.69	29.09	10.23	3.37	2.42	29.00	29.71	0.36	0.18	2284.56	1262.97
Yutian County	2842.70	1514.23	1.80	8.63	31.06	9.05	3.98	2.41	24.49	24.00	0.25	0.13	2135.02	1597.18
Hetian County	3813.75	1214.63	-4.69	8.04	18.25	8.51	3.07	2.81	31.53	27.61	0.24	0.12	1818.18	1595.71
Pishan County	2882.11	1306.94	2.18	7.65	19.95	5.96	4.10	2.58	26.00	26.19	0.24	0.13	1330.52	732.59
Minfeng County	2375.93	1371.57	4.61	7.20	33.29	9.56	3.86	1.29	15.75	15.28	0.19	0.10	3556.35	2816.85
Qiemo County	3391.81	1379.31	-1.90	7.40	70.80	37.19	6.16	3.44	35.45	25.82	0.19	0.09	4447.49	4231.44
Dazi County	4524.49	447.73	1.08	3.53	390.48	22.31	30.89	0.85	12.48	11.24	0.67	0.13	1259.83	794.26
Lei Wuqi County	4502.16	320.16	-1.34	2.71	568.95	17.23	67.28	5.21	37.97	28.26	0.71	0.12	1917.08	630.26
Linzhou County	4654.59	401.88	-1.00	3.25	421.63	22.68	31.13	2.61	19.63	16.89	0.61	0.15	1585.84	1209.35
Dangxiong County	4778.83	326.04	-2.35	3.10	424.80	37.94	30.41	2.87	33.55	30.85	0.58	0.15	1429.73	1395.41

Census Unit	DEM		TEM		GSpre		Wpre		Snow cover days		NDV		Travel time	
	Mean	STD	Mean	STD	Mean	STD	Mean	STD	Mean	STD	Mean	STD	Mean	STD
Bailang County	4663.06	350.10	-0.03	2.46	264.97	43.24	21.95	2.00	5.13	3.69	0.38	0.12	1004.74	578.00
Qushui County	4604.03	579.99	0.84	4.45	353.47	39.98	27.42	2.36	7.74	8.87	0.58	0.16	1479.74	1149.86
Sang Zhuzi District	4448.34	397.82	1.60	2.71	323.96	58.60	25.37	3.92	3.18	4.13	0.44	0.13	927.85	458.39
Jiangzi County	4685.45	323.45	-0.26	2.34	265.92	25.09	21.06	1.14	7.83	8.58	0.43	0.12	888.34	530.04
Naqu County	4739.78	235.92	-3.05	2.03	493.89	39.82	45.82	8.94	33.68	20.01	0.63	0.13	1738.90	1488.59
Jiali County	4983.99	230.68	-5.90	1.93	557.86	60.76	54.94	9.53	71.19	25.27	0.62	0.18	2986.68	1955.53
Dingqing County	4561.47	351.69	-3.84	3.44	607.14	24.90	69.49	4.79	66.36	35.65	0.69	0.15	1954.94	1157.27
Renbu County	4666.83	389.66	0.00	2.96	296.96	32.18	22.35	1.76	10.45	11.15	0.54	0.12	801.01	564.47
Changdong City	4546.87	371.28	-2.02	3.15	552.55	22.59	68.23	5.47	37.40	24.13	0.73	0.14	2212.08	864.25
Dong Longdeqing County	4743.11	493.19	-1.28	3.77	380.59	34.06	28.42	1.76	12.05	9.49	0.59	0.14	1336.14	1010.58
Chengguan District	3898.93	479.13	1.35	3.70	371.15	29.42	31.79	1.14	9.10	8.24	0.57	0.12	1014.16	613.86
Gongga County	4502.26	466.10	2.22	3.10	341.56	37.80	28.35	1.61	5.80	4.66	0.59	0.16	1158.97	689.06
Bianba County	4580.28	358.58	-2.57	3.05	638.90	19.38	73.13	4.23	56.83	41.78	0.71	0.17	1580.00	833.62
Naidong County	4622.30	508.54	0.25	3.80	333.33	22.84	30.25	2.57	14.89	13.35	0.58	0.16	1344.24	943.02
Nierong County	4862.57	196.69	-5.41	1.78	512.95	27.24	48.61	3.89	36.67	18.39	0.65	0.14	1904.45	1475.18
Nimu County	4835.86	433.94	-1.76	3.54	325.15	29.49	24.30	1.51	14.54	13.86	0.55	0.14	952.74	891.07
Nan Mulin County	4843.85	407.11	-2.00	3.15	328.54	39.96	25.19	2.37	13.95	11.63	0.52	0.14	1775.10	1408.22
Mo Zhugongka County	4786.07	376.35	-2.83	3.23	422.13	34.09	36.66	4.96	34.20	21.77	0.65	0.16	1711.52	1413.23
Mangkang County	4409.52	546.22	1.14	3.65	542.48	30.55	76.29	10.19	15.36	14.90	0.68	0.15	1870.72	938.94
Sangri County	4622.69	464.29	-0.55	3.86	363.42	30.44	32.00	4.65	21.58	18.21	0.65	0.14	1129.83	912.83
Lazi County	4582.14	361.90	0.50	2.47	292.61	73.00	54.16	13.74	3.26	4.05	0.40	0.12	1200.39	564.88
Jiangda County	4388.82	320.93	-1.70	3.06	548.85	20.01	67.63	4.66	36.03	24.25	0.76	0.11	1696.29	730.47
Dingjie County	4661.63	358.92	-0.53	2.52	319.27	73.15	78.48	22.52	9.53	15.89	0.26	0.12	1067.53	827.96
Biru County	4696.26	287.98	-3.78	2.56	616.93	49.83	67.29	9.30	57.16	32.86	0.68	0.15	1669.75	821.82
Suoxian County	4542.42	297.37	-2.64	2.34	633.94	21.81	72.20	5.00	51.74	29.73	0.75	0.13	1429.42	603.40
Baqing County	4734.24	234.24	-5.62	2.27	597.90	23.39	63.53	5.18	68.50	30.17	0.64	0.14	2814.20	1591.10
Gangba County	4868.37	267.99	-1.69	1.85	251.95	25.92	37.20	9.25	10.64	14.26	0.26	0.10	1187.92	720.11
Zhanang County	4595.28	518.68	1.08	3.76	344.81	23.54	29.86	1.02	10.45	10.35	0.60	0.16	1478.64	966.29
Lang Kazi County	4888.77	322.01	-1.92	2.52	296.70	21.98	28.76	3.24	14.64	13.31	0.43	0.13	920.09	770.78
Qusong County	4690.36	365.30	-0.57	2.85	314.30	15.11	30.87	3.64	18.70	11.22	0.53	0.14	998.37	635.91
Gongjue County	4350.66	322.81	0.11	2.55	544.72	17.04	67.70	3.20	19.27	10.60	0.72	0.11	1824.34	873.04
Chaya County	4412.53	391.04	0.07	2.97	537.64	14.56	68.38	3.35	20.94	14.98	0.71	0.13	2371.64	981.12
Sajia County	4701.95	384.32	-0.51	2.71	273.14	63.52	38.59	16.41	5.56	5.54	0.36	0.13	1113.03	693.91
Bayi District	4483.59	612.67	-2.02	4.15	590.44	74.07	70.54	10.11	53.89	27.98	0.62	0.19	2323.02	1256.88
Gong Bujiangda County	4847.52	360.58	-4.63	2.78	523.15	55.05	52.51	7.92	52.94	26.87	0.60	0.20	2779.55	1791.41
Langxian County	4408.82	523.64	0.20	3.97	380.99	37.85	38.64	6.83	24.87	27.32	0.71	0.16	1158.61	822.22
Jiacha County	4435.88	565.12	-1.10	4.66	352.64	47.77	31.55	7.30	25.61	23.33	0.72	0.18	1301.27	1298.62
Qiongjie County	4583.56	376.53	0.72	2.70	318.39	10.85	30.19	1.64	10.63	6.40	0.51	0.10	814.24	430.85
Milin County	4417.30	561.58	-1.33	4.03	503.93	64.77	58.48	9.08	43.85	28.12	0.68	0.18	1877.82	1502.48
Yadong County	4679.70	402.39	-0.78	2.39	307.11	91.96	39.51	22.43	29.05	21.44	0.39	0.24	992.69	623.61
Luolong County	4490.60	404.07	-1.00	3.46	592.25	14.47	71.39	4.73	41.56	35.59	0.65	0.14	1917.10	829.67
Longzi County	4465.12	605.60	0.37	3.68	320.84	110.86	36.30	13.77	22.17	23.72	0.53	0.20	983.63	574.05
Kangma County	4824.68	279.60	-1.45	2.03	247.72	13.90	22.66	2.06	13.37	15.22	0.26	0.11	810.06	498.93
Bomi County	4258.21	637.04	-2.49	4.62	618.99	46.70	82.04	5.21	67.83	35.13	0.63	0.23	2179.25	1157.74
Cuomei County	4842.92	253.30	-1.76	1.98	310.68	13.58	37.43	4.70	21.79	19.22	0.42	0.12	1151.32	718.54
Zuogong County	4573.86	582.55	-1.20	4.24	562.97	43.29	81.70	12.36	30.42	24.99	0.60	0.18	2463.69	1268.27
Xie Tongmen County	4951.87	318.49	-2.08	2.34	264.89	55.27	35.57	9.11	8.19	5.86	0.43	0.12	1569.00	1075.31
Basu County	4538.20	420.25	-3.11	3.85	563.37	18.78	74.59	6.39	50.41	33.38	0.60	0.18	1755.11	803.93
Dingri County	4738.82	352.22	-0.78	2.44	267.89	94.45	124.80	30.98	10.04	18.05	0.33	0.14	1272.90	738.29
Bange County	4855.10	212.29	-2.37	1.81	442.20	33.24	34.18	3.31	18.43	12.25	0.37	0.14	1953.81	1220.74
Luozha County	4637.34	486.99	-0.80	3.50	351.63	66.48	44.86	9.48	35.52	27.82	0.47	0.22	1306.71	956.52

Census Unit	DEM		TEM		GSpre		Wpre		Snow cover days		NDV		Travel time	
	Mean	STD	Mean	STD	Mean	STD	Mean	STD	Mean	STD	Mean	STD	Mean	STD
Nie Lamu County	4822.02	296.72	-1.01	2.11	183.79	44.80	154.08	17.03	19.60	17.18	0.31	0.12	1380.17	839.98
Andong County	4951.74	185.58	-6.48	2.19	399.08	101.29	31.73	8.61	30.26	16.83	0.36	0.15	6731.86	7037.81
Jilong County	4768.17	331.54	-0.92	2.53	154.52	57.54	147.02	11.42	28.42	22.80	0.35	0.13	1407.12	1195.85
Angren County	4958.25	321.45	-2.50	2.58	150.57	57.87	77.48	19.16	10.26	8.67	0.36	0.12	2326.59	1762.31
Chayu County	4242.69	708.29	1.64	4.70	692.00	209.93	120.71	28.00	47.46	27.04	0.55	0.18	1790.56	719.42
Shenzha County	4912.51	253.30	-2.05	2.33	369.69	47.54	38.54	4.12	14.06	7.36	0.34	0.13	1462.71	934.30
Saga County	4898.51	297.00	-2.00	2.48	116.43	24.75	127.66	15.92	16.84	11.13	0.35	0.11	1331.51	966.66
Cuoqin County	4986.18	320.26	-3.55	2.89	81.10	9.43	63.85	15.58	12.61	10.94	0.28	0.09	3221.53	2011.16
Zhongba County	4946.08	322.26	-3.96	2.89	112.19	39.85	91.61	35.48	31.17	27.44	0.29	0.10	2086.81	1466.08
Nima County	4947.91	227.94	-4.01	2.86	150.89	45.51	30.39	13.99	13.79	8.74	0.23	0.08	8275.43	8857.90
Cuona County	4565.97	1263.78	2.86	7.51	367.75	489.08	58.62	57.32	36.49	31.93	0.48	0.26	1439.23	1285.19
Gadong County	4914.34	364.74	-5.43	2.94	157.40	69.02	47.25	35.30	35.91	24.61	0.20	0.08	2271.64	1607.55
Pulan County	4865.01	318.54	-4.47	2.64	277.78	86.15	133.58	39.78	61.55	27.00	0.27	0.09	1392.38	791.68
Geji County	5006.95	293.27	-5.27	2.37	94.66	35.99	26.01	19.08	23.74	25.73	0.21	0.07	3505.01	2400.07
Zhada County	4647.50	363.13	-3.96	2.81	309.07	112.95	137.83	63.30	66.97	26.44	0.19	0.07	1414.32	1111.33
Motuo County	3707.74	1148.51	6.09	7.16	674.18	539.70	88.15	82.68	35.74	27.01	0.60	0.21	2309.28	1146.55
Ritu County	5073.28	323.16	-7.52	2.93	44.10	17.54	5.61	3.13	31.93	23.51	0.17	0.06	6859.54	4964.97
Gaize County	5035.83	233.07	-6.63	2.72	70.50	18.38	11.97	6.99	23.29	14.96	0.19	0.06	15013.82	7871.07
Shuanghu County	5001.08	178.32	-6.48	2.47	255.35	86.24	23.66	8.71	22.25	13.43	0.21	0.07	12724.85	7814.14
Ruo Donggai County	3603.68	155.10	0.73	0.99	636.38	14.52	83.73	4.62	22.85	9.00	0.84	0.06	1661.31	424.12
Maoxian County	4125.16	783.55	-1.05	4.84	738.02	27.86	107.73	5.79	54.27	33.22	0.65	0.17	2185.71	1210.03
Xiangcheng County	4421.47	466.12	-0.01	3.20	588.03	25.32	79.40	6.34	17.71	13.41	0.60	0.12	974.09	528.38
Hongyuan County	3762.40	220.47	0.13	1.67	660.94	18.19	91.63	4.52	41.38	18.30	0.83	0.07	2639.95	829.33
Heishui County	4243.14	598.14	-2.96	4.07	715.42	26.09	106.04	6.61	68.21	29.00	0.59	0.18	3830.43	1344.08
Songpan County	4083.24	306.97	-3.06	2.15	704.66	26.30	106.82	7.71	63.50	25.47	0.64	0.19	3513.23	1645.27
Xiaojin County	4382.69	263.54	-3.61	2.18	723.55	26.94	90.65	7.81	69.08	25.68	0.60	0.22	2072.40	794.67
Jiu Zhaigou County	3975.92	277.31	-2.42	1.81	685.51	18.79	102.24	6.17	49.25	18.77	0.64	0.18	3063.50	636.21
Aba County	3833.44	244.76	-0.13	1.75	686.01	15.29	89.55	2.44	30.48	13.15	0.83	0.06	1986.62	527.30
Jinchuan County	4303.31	458.12	-1.57	3.35	684.97	27.56	83.01	5.29	53.09	27.93	0.71	0.12	3634.58	1514.13
Jiulong County	4290.52	713.13	1.10	4.76	790.32	36.15	84.74	5.73	25.43	21.42	0.65	0.18	711.23	490.37
Luhuo County	4071.76	357.87	0.75	2.88	633.79	19.50	70.66	4.30	26.96	21.50	0.79	0.09	1361.63	719.43
Derong County	3999.91	744.06	4.72	4.50	571.30	45.05	95.45	18.55	7.66	8.17	0.62	0.11	845.35	308.12
Danba County	4206.06	746.76	-0.32	5.13	689.54	38.80	78.11	6.36	44.34	29.28	0.67	0.19	3492.47	2068.74
Ma Dongkang City	4164.32	407.27	-1.48	3.21	687.40	27.52	89.73	6.67	52.48	31.88	0.75	0.13	2027.24	646.93
Rangtang County	4172.83	304.26	-1.44	2.27	675.21	12.81	83.44	3.58	37.89	21.33	0.77	0.09	2122.14	769.57
Seda County	4265.18	229.85	-2.29	2.05	634.61	20.14	80.01	3.73	36.84	19.99	0.80	0.06	1729.58	735.09
Daocheng County	4447.44	428.73	-0.12	2.95	627.42	24.74	75.70	10.63	16.84	9.91	0.60	0.12	1270.97	477.13
Baiyu County	4443.20	325.59	-2.00	2.71	590.18	17.41	70.08	4.45	42.12	21.04	0.75	0.13	1762.04	1088.12
Yajiang County	4291.39	445.20	0.76	3.06	666.27	30.59	63.20	5.84	19.51	13.38	0.74	0.12	1065.69	919.16
Wenchuan County	4272.49	423.92	-3.38	3.01	751.92	17.66	101.50	6.37	60.02	30.08	0.47	0.21	2634.94	970.76
Lixian County	4338.14	307.78	-4.34	2.16	735.05	21.27	106.05	4.87	78.76	28.70	0.56	0.21	2341.58	834.69
Huding County	3814.65	968.02	6.77	5.99	788.64	57.80	89.66	11.26	17.85	21.19	0.61	0.17	1669.95	754.23
Litang County	4421.18	331.23	-0.52	2.73	611.46	32.32	62.63	6.04	24.36	17.19	0.70	0.13	1012.50	593.80
Daofu County	4228.95	404.97	-0.35	3.24	665.58	28.12	69.34	6.90	34.28	22.66	0.75	0.12	2836.63	1557.64
Batang County	4491.52	607.36	-1.09	4.36	570.90	18.05	71.77	6.15	30.50	22.79	0.64	0.17	1389.16	817.21
Xinlong County	4420.34	360.22	-1.99	3.04	627.82	19.90	68.85	6.42	38.61	23.20	0.72	0.14	2022.12	1199.76
Dege County	4326.18	332.27	-2.55	2.90	575.61	24.68	72.37	6.10	45.48	28.19	0.78	0.15	1228.89	540.24
Shiqu County	4474.74	243.72	-4.51	2.60	528.83	27.27	66.85	5.37	56.32	29.98	0.75	0.10	1845.12	864.86
Ganzi County	4286.80	297.42	-1.96	2.76	611.91	18.70	76.10	3.19	41.59	24.20	0.79	0.09	1504.80	731.88
Huangzhong County	3176.61	353.86	-0.98	2.31	504.99	35.51	50.30	4.87	22.12	7.26	0.79	0.09	314.85	166.38
Xun Huasalazu Autonomous County	3040.78	673.15	1.53	3.63	502.82	56.85	54.24	8.40	14.81	10.16	0.65	0.18	377.30	246.73

Census Unit	DEM		TEM		GSpre		Wpre		Snow cover days		NDV		Travel time	
	Mean	STD	Mean	STD	Mean	STD	Mean	STD	Mean	STD	Mean	STD	Mean	STD
Huangyuan County	3412.47	330.05	-2.35	2.24	502.97	22.81	50.01	3.85	26.87	12.30	0.77	0.07	424.17	260.18
Tongde County	3738.61	336.42	-1.57	2.08	506.84	51.39	53.20	8.53	17.38	10.23	0.76	0.11	789.69	382.40
Hua Longhuizu Autonomous County	2835.76	463.21	2.46	2.75	468.21	40.49	47.93	5.71	9.93	5.34	0.62	0.16	514.70	316.28
Ledong District	2710.17	533.68	2.40	3.43	429.99	60.98	44.77	7.74	15.73	10.80	0.68	0.13	319.55	236.81
Guinan County	3390.72	385.83	-0.12	2.44	477.04	49.62	49.07	7.80	15.65	9.51	0.64	0.18	440.21	144.26
Hu Zhutuzu Autonomous County	3219.28	485.10	-1.17	3.39	480.80	53.42	50.98	9.40	29.84	22.30	0.74	0.13	415.93	295.27
Jianzha County	3147.15	494.99	1.11	2.78	498.38	38.98	51.33	5.56	11.71	5.66	0.70	0.14	337.61	151.67
Zeku County	3785.32	205.70	-2.45	1.51	561.15	27.99	62.57	5.48	23.03	8.94	0.80	0.06	764.66	389.49
Pingan District	2774.37	430.57	2.40	2.76	447.37	50.99	43.74	7.19	12.40	5.73	0.66	0.15	181.80	139.46
He Nanmengguzu Autonomous County	3740.78	181.98	-1.52	1.05	612.51	24.58	71.83	5.17	23.97	8.02	0.84	0.05	810.35	396.47
Men Yuanhuizu Autonomous County	3473.96	353.06	-4.19	2.53	505.60	24.65	56.46	6.96	48.36	34.21	0.73	0.15	802.27	561.31
Gangcha County	3696.68	277.29	-5.17	2.60	436.36	37.44	39.79	4.63	29.60	17.81	0.73	0.11	963.03	530.25
Haiyan County	3483.71	282.36	-3.43	2.40	486.73	27.57	49.32	4.95	30.72	19.34	0.73	0.15	513.70	369.06
Tongren County	3520.23	384.15	-0.64	2.21	559.73	36.24	61.50	5.99	17.34	7.54	0.78	0.11	733.06	384.85
Chengzhong District	2550.51	97.52	3.98	0.55	427.44	11.28	39.73	2.06	8.27	1.66	0.63	0.07	207.04	71.49
Xinghai County	3893.68	470.71	-3.44	3.61	417.26	34.03	44.25	8.24	22.88	18.83	0.66	0.16	786.85	425.46
Jiuzhi County	4118.66	223.61	-3.16	1.49	670.19	22.50	86.52	4.69	37.99	15.43	0.82	0.06	1899.23	580.82
Banma County	4228.23	246.32	-2.98	1.77	653.80	26.02	84.51	3.61	38.15	18.85	0.79	0.06	1894.85	692.40
Gonghe County	3399.67	411.60	-0.87	3.11	385.01	54.03	37.18	6.74	12.35	9.57	0.54	0.21	772.75	520.43
Guide County	3145.83	479.21	1.17	3.12	485.35	42.69	49.78	6.74	12.91	10.82	0.55	0.20	429.07	228.28
Yushu City	4476.50	268.02	-3.71	2.47	549.47	22.86	61.80	5.22	40.38	22.00	0.76	0.11	1834.54	805.19
Nangqian County	4330.34	277.37	-0.93	2.42	557.58	26.32	60.69	4.37	26.76	19.26	0.75	0.09	1708.46	737.20
Qilian County	3752.92	360.42	-7.27	1.95	430.69	52.73	42.28	6.99	43.30	22.86	0.65	0.19	796.24	466.00
Gande County	4251.77	190.85	-4.54	1.71	573.58	33.13	72.54	4.59	41.89	19.38	0.79	0.07	1539.58	713.99
Wulan County	3447.07	442.20	-1.86	3.75	257.81	61.96	32.34	6.85	23.41	19.75	0.37	0.17	981.05	342.45
Chengdong County	4547.66	179.33	-6.11	1.85	490.65	38.26	59.89	5.75	52.08	19.65	0.68	0.12	3113.19	2141.88
Chengbei District	2483.29	111.55	4.38	0.63	414.15	18.61	37.75	3.09	9.89	1.64	0.65	0.08	131.60	38.59
Maqin County	4252.12	313.39	-5.16	2.51	516.91	43.31	64.47	6.95	45.73	27.48	0.75	0.12	1665.16	765.32
Chengdong District	2468.04	133.74	4.95	0.60	409.06	18.80	36.94	2.84	8.15	1.33	0.61	0.08	119.55	44.34
Tianjun County	3983.53	287.31	-7.62	2.17	352.17	46.87	36.50	5.22	34.68	22.10	0.55	0.18	1477.46	914.01
Donglan County	3923.86	647.68	-3.95	4.65	262.65	94.41	36.94	13.10	39.62	28.51	0.36	0.15	1792.81	1005.48
Zadong County	4745.53	213.72	-5.48	1.79	520.40	57.98	51.59	10.11	37.55	22.94	0.58	0.14	2302.21	1347.68
Dari County	4410.63	192.53	-5.28	1.75	556.09	44.09	72.32	5.59	56.48	25.32	0.73	0.10	2090.36	1390.18
De Lingha City	3878.28	481.62	-6.33	4.18	241.07	54.36	31.64	7.46	39.97	24.37	0.31	0.12	1606.31	943.94
Qu Malai County	4558.25	157.01	-5.96	1.42	346.42	67.26	36.16	10.07	37.24	16.33	0.44	0.18	3155.25	1360.80
Zhidong County	4738.28	208.71	-6.59	1.90	328.49	96.65	29.19	12.70	34.32	18.33	0.39	0.20	4077.16	2532.64
Madong County	4401.85	154.20	-6.27	1.40	393.62	40.14	51.86	6.27	44.09	17.21	0.53	0.14	1505.74	786.13
Chengxi District	2500.74	99.21	4.62	0.65	414.45	14.65	36.76	2.19	8.43	1.39	0.59	0.07	132.70	35.58
Da Chaidanlenghumangyaxingzheng District	3785.73	627.95	-4.45	4.53	98.51	59.19	10.25	9.01	33.85	29.15	0.18	0.09	1731.86	1086.26
Ge Dongmu City	4589.70	620.62	-5.84	3.26	270.88	114.28	21.96	8.92	31.43	19.54	0.31	0.13	3773.10	3366.67
Gulang County	2296.93	257.81	4.12	1.30	264.15	37.79	32.49	4.48	23.59	6.62	0.50	0.10	346.65	185.39
Hezuo City	3442.02	241.63	-0.10	1.35	577.70	14.66	68.48	3.50	23.87	9.17	0.82	0.05	408.83	206.95
Luqu County	3605.42	199.52	-0.19	1.04	625.81	17.45	76.13	4.13	22.40	8.01	0.84	0.05	1432.21	360.13
Maqu County	3800.24	298.36	-1.10	2.23	661.65	21.72	82.93	4.74	28.55	12.25	0.84	0.06	1937.31	613.11
Xiahe County	3497.53	313.16	-0.24	1.69	589.14	27.29	67.75	5.08	19.54	7.90	0.82	0.07	750.61	495.56
Su Nanyuguzu Autonomous County	3162.29	727.85	-3.02	4.77	349.64	83.28	36.82	7.89	34.30	25.43	0.46	0.20	1430.75	619.27

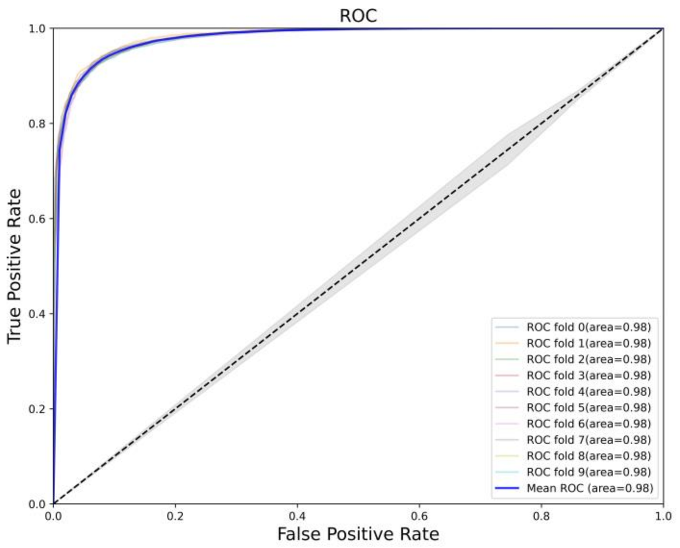

Supplementary Fig.S1 Ten-fold cross-validation results of the random forest classification model.

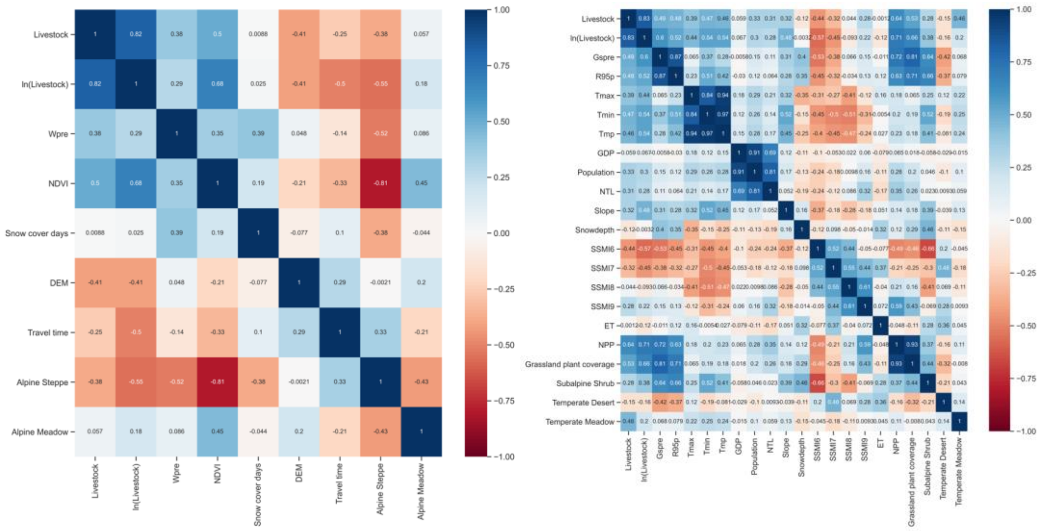

Supplementary Fig.S2 Correlation between livestock density and covariates.

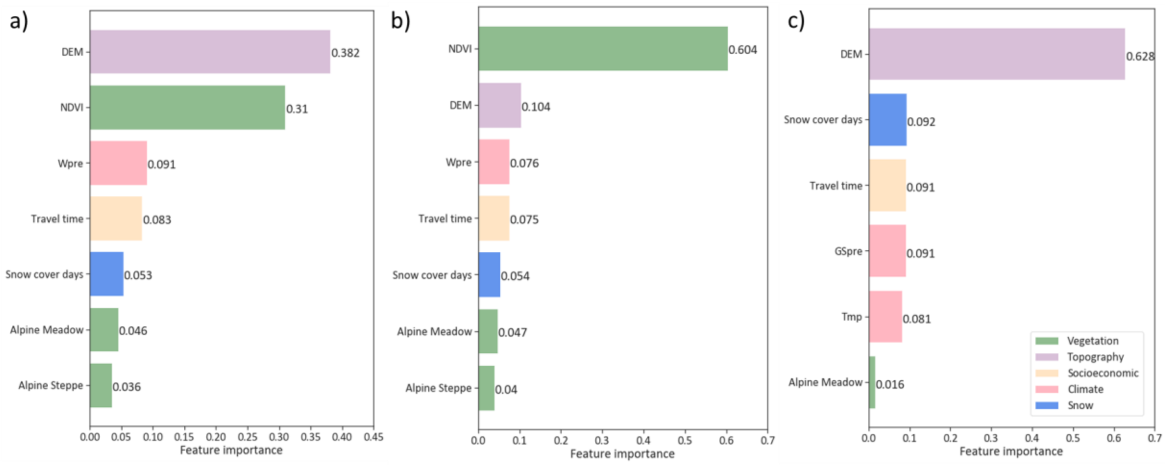

Supplementary Fig.S3 a) Contribution of covariates to livestock density estimation; b) Contribution of covariates to cattle density estimation; c) Contribution of covariates to sheep density estimation.

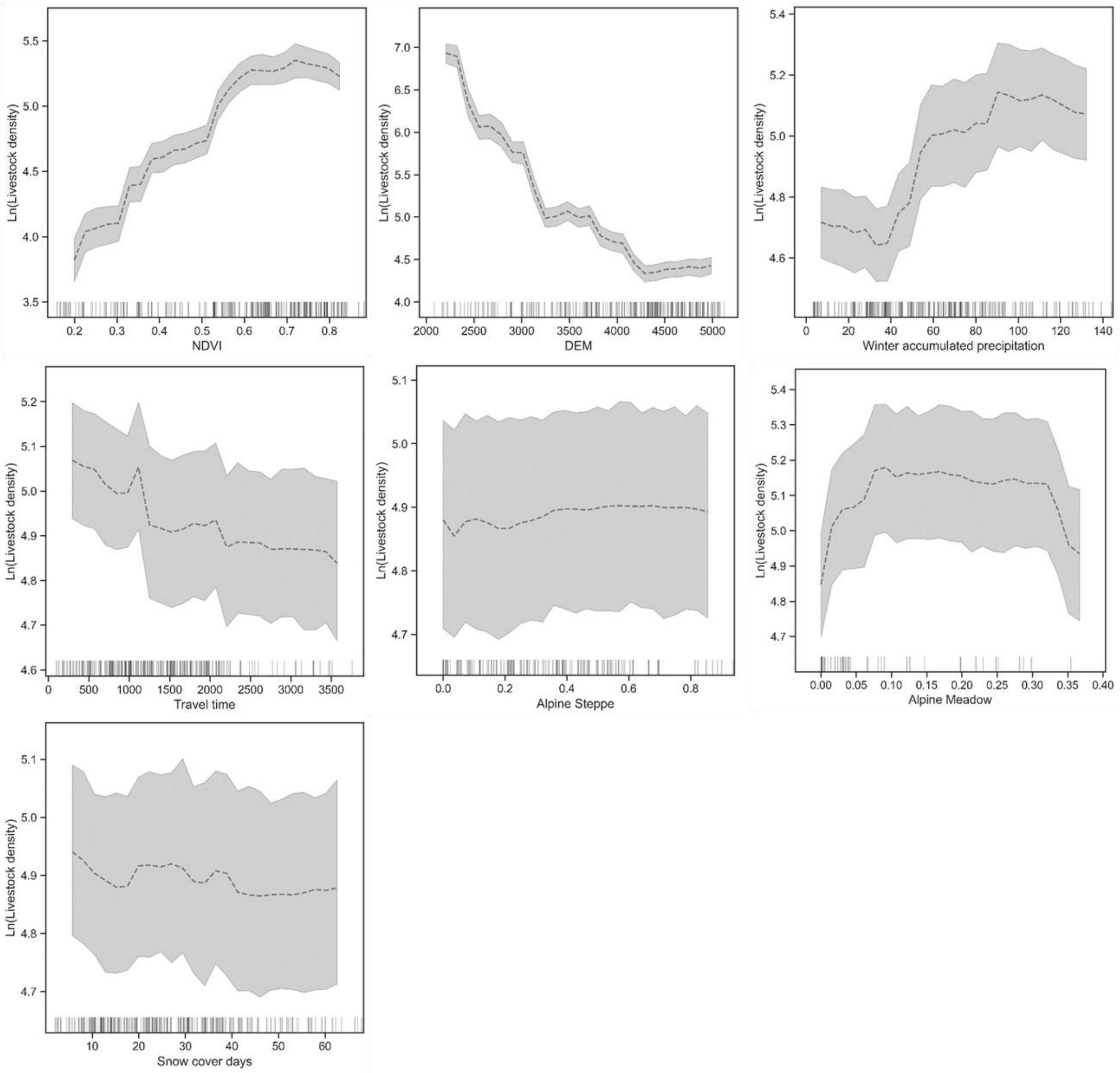

Supplementary Fig.S4 Response of covariates to livestock densities given by the partial dependence plot.

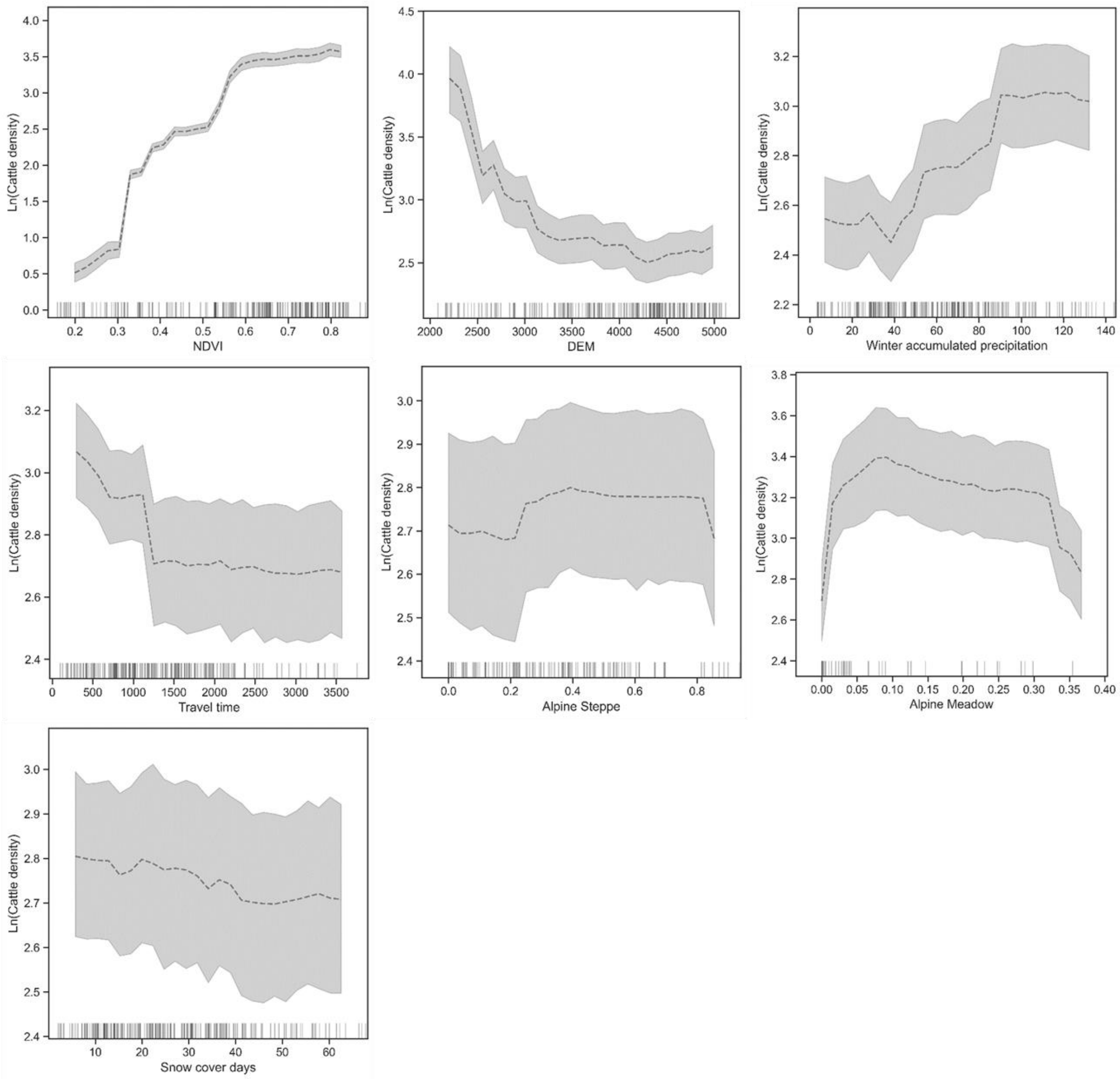

Supplementary Fig.S5 Response of covariates to cattle density given by the partial dependence plot.

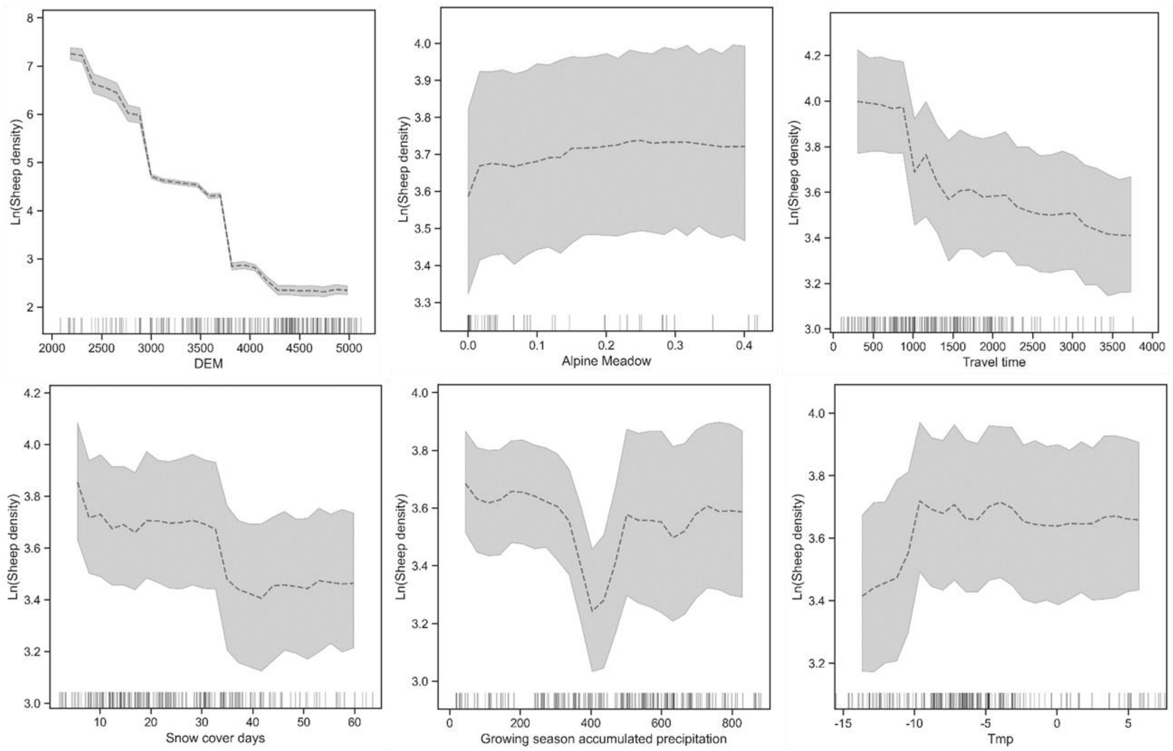

Supplementary Fig.S6 Response of covariates to sheep density given by the partial dependence plot.

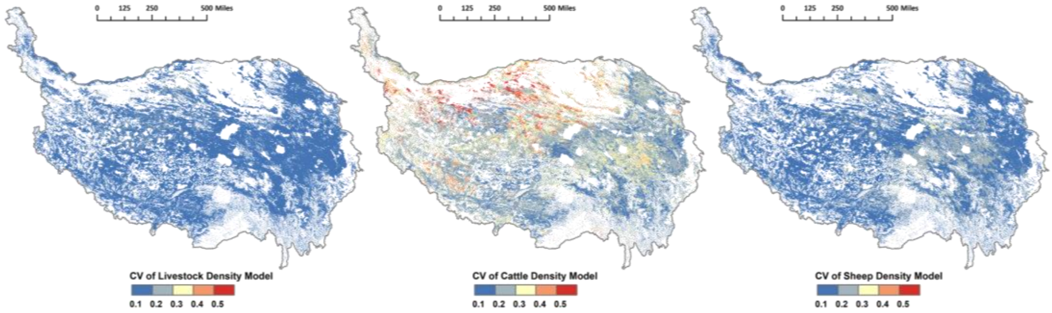

Supplementary Fig.S7 Coefficient of variation (CV) of livestock, cattle, and sheep density model (The CV of the ten-fold internal cross-validation results).

References

1. Earth Resources Observation and Science (EROS) Center. USGS EROS Archive - Digital Elevation - Shuttle Radar Topography Mission 1 Arc-Second Global. (2018) doi:/10.5066/F7PR7TFT.
2. Fischer, G. . *et al.* Global Agro-ecological Zones Assessment for Agriculture (GAEZ). *IIASA, Laxenburg, Austria FAO, Rome, Italy* (2008).
3. Peng, S., Ding, Y. & Li, Z. 1-km monthly temperature and precipitation dataset for China from 1901–2017. *Earth Syst. Sci. Data* **11**, 1931–1946 (2019).
4. Wu, J. & Gao, X. A gridded daily observation dataset over China region and comparison with the other datasets. *Chin. J. Geophys* **56**, 1102–1111 (2013).
5. Running, S., Mu, Q. & Zhao, M. MOD16A2 MODIS/Terra Net Evapotranspiration 8-Day L4 Global 500m SIN Grid V006. (2017) doi:10.5067/MODIS/MOD16A2.006.
6. Che, T. & Dai, L. Long-term series of daily snow depth dataset in China (1979-2020). *National Tibetan Plateau Data Center* (2015) doi:10.11888/Geogra.tpdc.270194.
7. Zheng, Z. & Cao, G. Snow cover dataset based on multi-source remote sensing products blended with 1km spatial resolution on the Qinghai-Tibet Plateau (1995-2018). *National Tibetan Plateau Data Center* (2019) doi:10.11888/Snow.tpdc.270102.
8. Running, S., Mu, Q. & Zhao, M. MOD17A2H MODIS/Terra Gross Primary Productivity 8-Day L4 Global 500m SIN Grid V006 [Data set]. *NASA EOSDIS L. Process. DAAC* (2015) doi:10.5067/MODIS/MOD17A2H.006.
9. Running, S., Mu, Q. & Zhao, M. MOD17A3H MODIS/Terra Net Primary Production Yearly L4 Global 500m SIN Grid V006 [Data set]. *NASA EOSDIS L. Process. DAAC* (2015) doi:10.5067/MODIS/MOD17A3H.006.
10. Didan, K. MOD13Q1 MODIS/Terra Vegetation Indices 16-Day L3 Global 250m SIN Grid V006. (2015) doi:10.5067/MODIS/MOD13Q1.006.
11. Lehnert, L. W. *et al.* Retrieval of grassland plant coverage on the Tibetan Plateau based on a multi-scale, multi-sensor and multi-method approach. *Remote Sens. Environ.* **164**, 197–207 (2015).
12. Zhang, X. Vegetation map of the People's Republic of China (1:1 000 000). *Geol. Publ. House (GPH)* (2007).
13. Yi, L., Xiong, L. & Yang, X. Method of Pixelizing GDP Data Based on the GIS. *J. Gansu Sci.* **18**, 54–58 (2006).
14. Elvidge, C. D., Zhizhin, M., Ghosh, T., Hsu, F. C. & Taneja, J. Annual time series of global viirs nighttime lights derived from monthly averages: 2012 to 2019. *Remote Sens.* **13**, 1–14 (2021).
15. Oak Ridge National Laboratory. LandScan Global Population Database 2019. (2020).
16. Weiss, D. J. *et al.* A global map of travel time to cities to assess inequalities in accessibility in 2015. *Nature* **553**, 333–336 (2018).
